# Preclinical model in HCC: the SGK1 kinase inhibitor SI113 blocks tumor progression *in vitro* and *in vivo* and synergizes with radiotherapy

**DOI:** 10.18632/oncotarget.5527

**Published:** 2015-10-08

**Authors:** Cristina Talarico, Lucia D'Antona, Domenica Scumaci, Agnese Barone, Francesco Gigliotti, Claudia Vincenza Fiumara, Vincenzo Dattilo, Enzo Gallo, Paolo Visca, Francesco Ortuso, Claudia Abbruzzese, Lorenzo Botta, Silvia Schenone, Giovanni Cuda, Stefano Alcaro, Cataldo Bianco, Patrizia Lavia, Marco G. Paggi, Nicola Perrotti, Rosario Amato

**Affiliations:** ^1^ Department of “Scienze della Salute”, University “Magna Graecia” of Catanzaro, Viale Europa, Catanzaro, Italy; ^2^ Department of “Medicina Sperimentale e Clinica”, University “Magna Graecia” of Catanzaro, Viale Europa, Catanzaro, Italy; ^3^ Section of Pathology, Regina Elena National Cancer Institute, IRCCS, Rome, Italy; ^4^ Experimental Oncology, Regina Elena National Cancer Institute, IRCCS, Rome, Italy; ^5^ Department of Biotecnologie, Chimica e Farmacia, University of Siena, Siena, Italy; ^6^ Department of Farmacia, University of Genova, Genova, Italy; ^7^ Institute of Molecular Biology and Pathology (IBPM), National Research Council of Italy (CNR), c/o University “La Sapienza”, Rome, Italy

**Keywords:** SGK1, HCC, kinase inhibitor, radiotherapy, apoptosis

## Abstract

The SGK1 kinase is pivotal in signal transduction pathways operating in cell transformation and tumor progression. Here, we characterize in depth a novel potent and selective pyrazolo[3,4-d]pyrimidine-based SGK1 inhibitor. This compound, named SI113, active *in vitro* in the sub-micromolar range, inhibits SGK1-dependent signaling in cell lines in a dose- and time-dependent manner. We recently showed that SI113 slows down tumor growth and induces cell death in colon carcinoma cells, when used in monotherapy or in combination with paclitaxel. We now demonstrate for the first time that SI113 inhibits tumour growth in hepatocarcinoma models *in vitro* and *in vivo*. SI113-dependent tumor inhibition is dose- and time-dependent. *In vitro* and *in vivo* SI113-dependent SGK1 inhibition determined a dramatic increase in apotosis/necrosis, inhibited cell proliferation and altered the cell cycle profile of treated cells. Proteome-wide biochemical studies confirmed that SI113 down-regulates the abundance of proteins downstream of SGK1 with established roles in neoplastic transformation, e.g. MDM2, NDRG1 and RAN network members. Consistent with knock-down and over-expressing cellular models for SGK1, SI113 potentiated and synergized with radiotherapy in tumor killing. No short-term toxicity was observed in treated animals during *in vivo* SI113 administration. These data show that direct SGK1 inhibition can be effective in hepatic cancer therapy, either alone or in combination with radiotherapy.

## INTRODUCTION

Hepatocellular carcinoma (HCC) is a major health problem, accounting for more than 782,000 new cases per year worldwide [1, 2]. The disease is generally diagnosed at advanced stages and lacks effective treatment [3]. Therefore, it is important to elucidate molecular mechanisms that can be used to identify pharmacological targets for the treatment of HCC [4]. Increasing efforts have been recently devoted to the development of radiotherapy-based approaches for conservative or rescue treatment of HCC [5]. Focal irradiation and/or resection are indicated in very early to intermediate stages. HCC patients unsuitable for conservative therapies are then ideal candidates for experimental, combined radio-biological approaches.

The serum- and glucocorticoid-regulated kinase 1 (SGK1) is a member of the AGC (protein A, G, C, families) kinase group, and shares structural and functional similarities with the AKT family of kinases [6]. mTORC2 phosphorylates the SGK1 hydrophobic motif (H-motif) on serine 422 [7], thus priming the kinase for phosphorylation by 3-phosphoinositide-dependent kinase-1 (PDK1) [8]. SGK1 is regulated by insulin, IGF-1, cAMP, glucocorticoids [9–12] and IL-2 [13] and transduces survival signals in normal and cancer cells. Increased SGK1 expression has been found in several human tumors, including prostate carcinoma [14], non-small cell lung cancer [15] and HCC [16–18]. Conversely, SGK1 knock-out models were shown to be resistant to chemically-induced colon carcinogenesis [19]. SGK1 regulates cell survival, proliferation and differentiation via phosphorylation of Mouse Double Minutes 2 (MDM2), which leads to the ubiquitylation and proteosomal degradation of p53 [20]. SGK1 also activates transcription of RANBP1, a major regulator of the RAN GTPase, thus affecting mitotic stability and paclitaxel sensitivity in colon carcinoma cells [21]. Finally, SGK1 activates N-Myc down-regulated gene 1 (NDRG1), by catalysing its phosphorylation on threonine 346. The NDRG1 factor is considered to be a specific SGK1 substrate [22]. However, in some breast cancer cells lines displaying low/undetectable levels of SGK1, and sensitive to Akt1 specific inhibitors, a marked phosphorylation of NDRG1 was detected, that was suppressed by Akt inhibitors, differently from resistant cells [23], suggesting that under certain conditions Akt can also phosphorylate SGK1 targets. NDRG1 has an important role in cell proliferation and differentiation [24, 25] and a debated role in oncogenesis, as it mainly behaves like an oncosuppressos in several tumor models [26, 27], yet appears to be a key determinant of resistance towards alkylating chemotherapy in malignant gliomas [28] and is associated with aggressive tumor behavior in HCC [29], where NDRG1 suppression induces apoptosis [30].

We recently identified a small molecule, SI113, that is particularly effective in inhibiting SGK1 kinase activity, while being much less effective on AKT1, ABL and SRC activities [31, 32]. SI113 was able to induce cell death in various malignant cell lines, including MCF7 breast carcinoma, A-172 malignant glioma and RKO colon carcinoma and synergized with paclitaxel in induction of apoptosis [31]. We now present *in vitro* data obtained in HepG2 and HuH-7 cell lines, as well as *in vivo* data from HCC xenografts in NOD/SCID mice, indicating that SI113 inhibits liver cancer cell proliferation, induces apoptosis and necrosis and potentiates the effects of radiotherapy, mimicking some of the effects of SGK1 knock-down. Based on the apparent lack of toxicity and the consistent effectiveness of SI113 in mice, this molecule is of potential value in the treatment of human HCC, either alone or in combination with radiotherapy.

## RESULTS

### SI113, a new inhibitor of SGK1, strongly reduces cell viability in HCC cells

**Figure 1:**
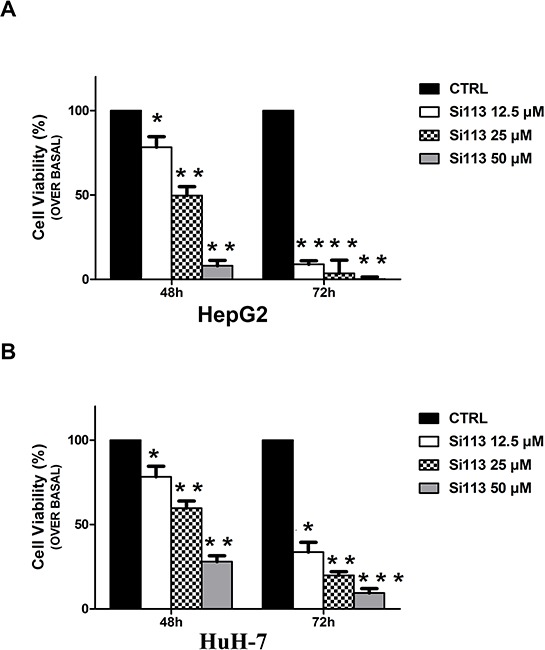
Cell Growth inhibition induced by SI113 in HepG2 and HuH-7 in human HCC cell lines **A.** HepG2 human liver hepatocellular carcinoma cell line. **B.** HuH-7 human liver hepatocellular carcinoma cell line. The histograms represent the number of cells treated with SI113 (12.5, 25 or 50 μM) for 48 and 72 h and are expressed as percentage of the number of control cells (HepG2 ctrl 48 h 89453 ± 4527, 72 h 500523 ± 46423; HuH-7 ctrl 48 h 92787 ± 3378, 72 h 145333 ± 13889) treated with vehicle alone at 48 and 72 h. Results represent the mean ± S.E. of six independent experiments for each cell line.

HepG2 and HuH-7 cell lines were plated (see Methods section). After 24 h, when cells were approximately 60% confluent, SI113 was added in increasing concentrations (12.5, 25 and 50 μM), and cell viability was estimated after 48 and 72 h. In both cell lines, SI113 yielded a significant time- and dose-dependent reduction in the number of viable cells (Figure 1, panel A and B). All subsequent experiments were performed using 12.5 μM SI133, unless otherwise indicated.

### SI113 inhibits cell cycle progression and induces apoptosis in HuH-7 and HepG2 cell lines in a time-dependent manner

**Figure 2:**
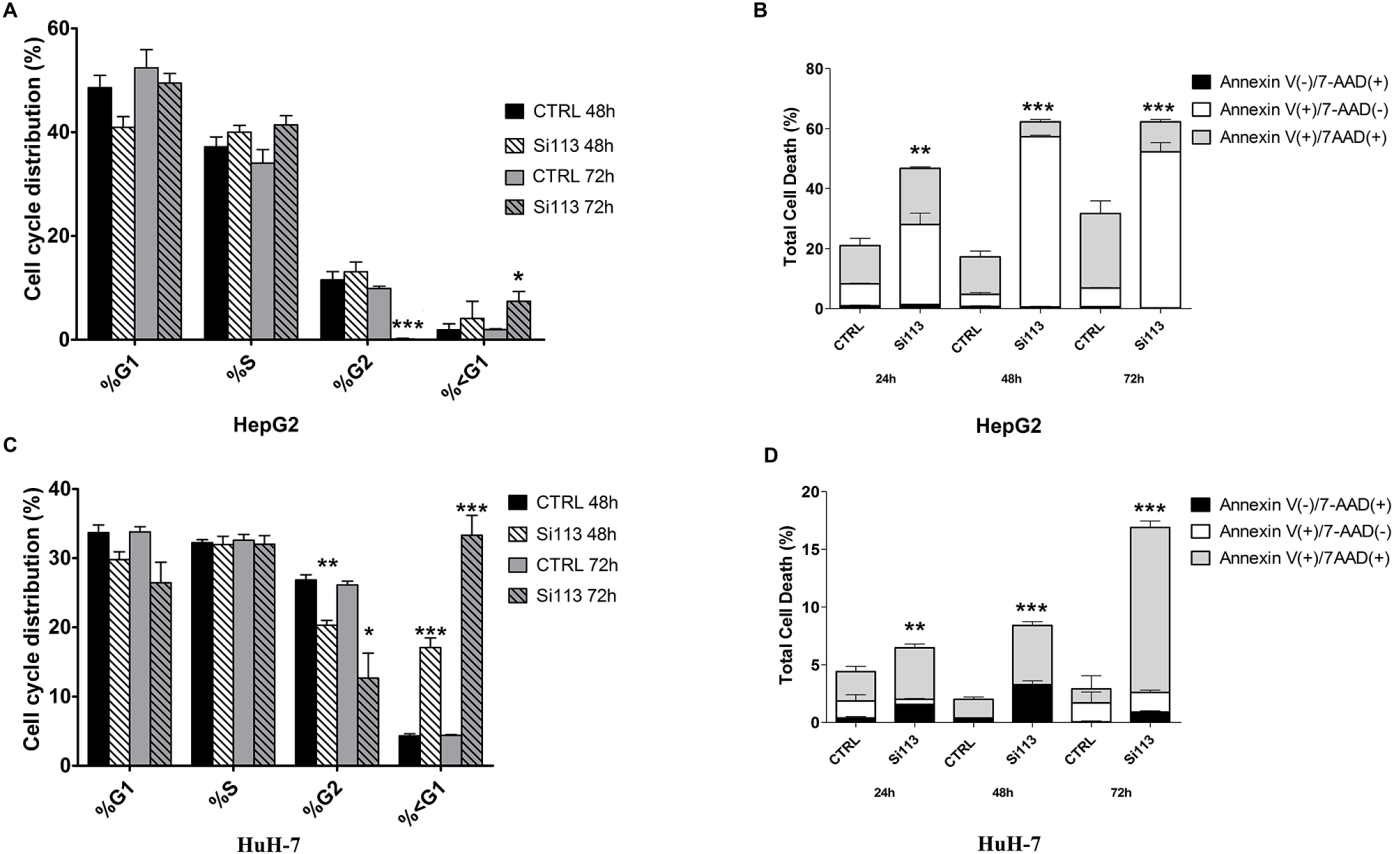
Time course of SI113 induced cell cycle regulation and necro/apoptosis in HepG2 and HuH-7 Histograms represent cell cycle distribution of HCC cell lines treated with vehicle alone or SI113 (12.5 μM) for 48 or 72 h. **A.** HepG2 cell line. **C.** HuH-7cell line. Data are the average ± S.D. of three independent experiments. **B.** HepG2 cell line and **D.** HuH-7 cell line were treated with SI113 (12.5 μM) or vehicle alone for 24, 48 and 72 h. The percentage of cells stained with either Annexin V, or 7-ADD, or both (calculated using the Guava Annexin assay) is shown in the graph, representing the average of three independent experiments. Histograms depict the percentage of early apoptotic Annexin V(+)/7-AAD(−), late apoptotic Annexin V(+)/7−AAD(+) and necrotic/dead Annexin V(−)/7-AAD(+) cells, after exposure to SI113, as indicated. Data are the average ± S.E. of three independent experiments.

We used flow cytometry to assess whether SI113 affected cell cycle progression. SI113 inhibited cell cycle progression in both HepG2 and HuH-7 cell systems. In HepG2, a significant reduction of cell population in the G2/M phase was observed after 72 h of treatment, concomitant with a significant increase in the percentage of <G1 hypodiploid cells (Figure 2, panel A). In HuH-7 cells, the effect of SI113 was already evident after 48 h and more significant at 72 h (Figure 2, panel C, Supplementary File S2 for quantitative data and significance, and Supplementary Figure S1–S4 for cell cycle and Guava graphs). HepG2 and HuH-7 cells treated with SI113 for 24, 48 and 72 h were also analyzed by Guava Nexin Assay. A significant increase in total cell death was demonstrated in SI113-treated cells at each time point in both HepG2 and HuH-7cultures (Figure 2, panel B and D).

In particular, in HepG2 cells (Figure 2, panel B), treatment with SI113 for 24 h yielded a significant increase in early apoptotic cells, which gradually became more significant as the treatment was prolonged to 48 h and 72 h. In HuH-7cells treated with SI113 (Figure 2, panel D), late apoptotic cells increased progressively after 48 and 72 h. All differences with the corresponding controls were highly significant, as indicated in Figure Legends and Supplementary File S2. These data indicate therefore that SI113 gradually suppressed the fraction of cycling cells and concomitantly activated the apoptotic response pathways over time. These results were further confirmed in Guava-based multi caspase assays and Western blot for active caspase 3, which showed a clear pro-apototic effect of SI113 (Supplementary Figure S5).

### Evidence for selectivity of SGK1 targeting by SI113

**Figure 3:**
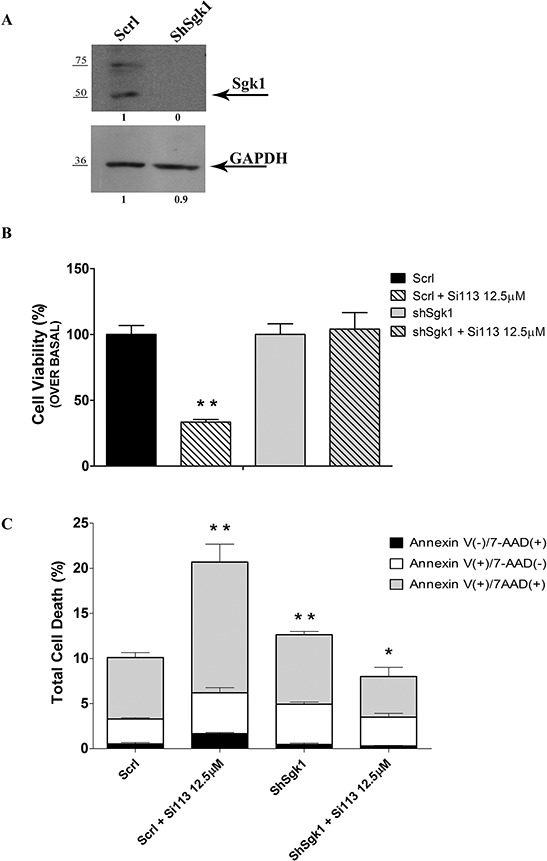
Target specificity for SI113 **A.** SGK1 expression in ShSCRLHuH-7 cells (left) and Sh*SGK1*HuH-7 cells (right). GAPDH was used as a loading control. **B.** HuH-7cell line. The histograms represent the number of Sh*SCRL* and sh*SGK1* cells treated for 72 h with either SI113 (12.5 μM) or vehicle alone. Results are expressed as the percentage relative to the baseline (Scrl 571333 ± 38456; ShSGK1 557333 ± 44779) in either cell line treated with vehicle alone. Results represent the mean ± S.E. of three independent experiments.*P ≤ 0.05; **P ≤ 0.01; ***P ≤ 0.001. **C.** ShSCRL and ShSGK1 HuH-7 cell lines were treated with SI113 (12.5 μM) or vehicle alone for 72 h. Percentages of cells stained with either Annexin V, or 7-ADD, or both (calculated using the Guava Annexin assay) are represented in the graphs. Histograms depict the percentage of cells in early apoptosis, late apoptosis, or necrosis/death after exposure to SI113, as indicated. Data are the average ± S.E. of three independent experiments. The percentage of apoptotic cells in vehicle treated ShSGK1 HuH-7 cells (third column) is compared with vehicle treated ShSCRL cells (first column). The percentage of SI113 treated ShSGK1 HuH-7 cells (fouth column) is compared with vehicle treated ShSGK1 HuH-7 cells (third column). The percentage of apoptotic cells in SI113 treated ShSGK1 HuH-7 cells (fourth column) is not significantly different from the percentage of apoptotic cells in vehicle treated ShSCRL cells. *P ≤ 0.05; **P ≤ 0.01; ***P ≤ 0.001.

To verify the functional SGK1 specificity of SI113-dependent effects on cell proliferation and survival, we produced HuH-7 cell clones stably expressing either the shRNA for *SGK1* (Sh*SGK1-*HuH-7cells) or a scrambled shRNA (Scrl-HuH-7cells). SGK1 silencing was verified by Western blot using anti-SGK1 antibodies (Figure 3, panel A). Considering the low proliferation rate and increased apoptosis typically observed in SGK1-silenced cell lines [20], different cell numbers were plated. ScrlHuH-7 (1.5 × 10^4^ cells) and Sh*SGK1* (2.5 × 10^4^ cells) were plated and, after 24 h (when approximately 60% confluent), SI113 or vehicle were added. Cell proliferation and viability were estimated after 72 h, when both vehicle-treated cultures had reached comparable cell numbers (ScrlHuH-7 cultures: 571333 ± 38456 cells; Sh*SGK1-*HuH-7 cultures, 557333 ± 44779 cells). We found that SI113 did not affect the viability of Sh*SGK1* cells (dashed right histogram), while significantly affecting that of Scrl-HuH-7 cells, in which the number of viable cells decreased to 30% upon SI113 treatment (*P* = 0.008) (Figure 3, panel B).

Stable Scrl or sh*SGK1*-expressing HuH-7 cells treated with SI113 for 72 h were also Guava Nexin assayed (Figure 3, panel C). The early apoptosis fraction significantly increased in Sh*SGK1-*HuH-7 compared with Scrl-HuH-7 cells, consistent with previous observations [21]. SI113 treatment for 72 h increased all stages of cell death, i.e. early apoptotic, late apoptotic and necrotic/dead cells, selectively in Scrl-HuH-7. SI113 had instead no pro-apoptotic effect in Sh*SGK1-*HuH-7cells, in which a modest decrease in late apoptotic cells - if any - was recorded compared with vehicle-treated controls. All differences with the corresponding controls were significant and are indicated in Figure Legends and Supplementary File S2. Taken together, these data strongly suggest that SGK1 function is necessary for SI113 to elicit its pro-apoptotic effect.

### Identification of differentially expressed proteins in cells treated with SI113

We recently identified several proteins either down-regulated or up-regulated upon *SGK1* specific RNA silencing. Among those, we focused on the RAN/RANBP1 network, which pointed to SGK1 as a key regulator of mitotic stability and paclitaxel resistance [21, 31]. We now extended our proteomic analysis to HCC cells treated for 72 h with either SI113 or vehicle alone.

**Figure 4:**
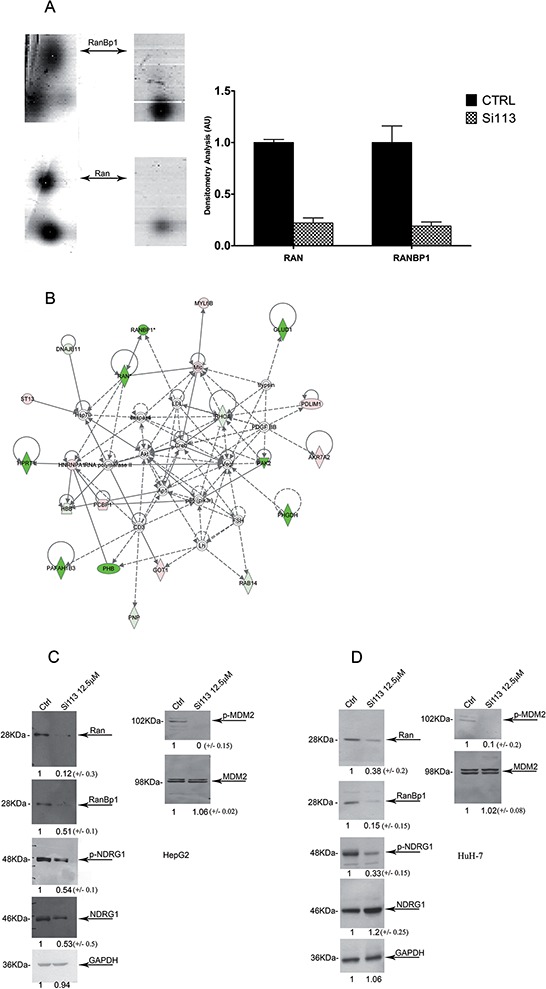
The addition of SI113 induces the inhibition of p-NDRG1/NDRG1 and p-MDM2/MDM2 and downregulates the RAN/RANBP1 axis **A.** RANBP1 and RAN spots in 2DE gels from HuH-7 cells untreated (left lanes) and treated (right lanes) with 12.5 μM SI113 for 72 h. 2DE tables for the identified proteins with specific values under treatment vs. control are shown in Supplementary Figure S2 (right panel). Signal intensity values (mean ± S.D.) for treated and untreated HuH-7 cells were calculated from RANBP1 and RAN densitometric scanning from three independent 2DE gel experiments. **B.** Functional pathway analysis (IPA software). Network analysis of protein expression was performed using the Ingenuity software. Each network displays the protein products as nodes (different shapes representing the functional classes of proteins) and the biological relationships between the nodes as lines. The length of each line reflects the amount of literature evidence supporting the node-to-node relationship. The color intensity of each node indicates the degree of up-regulation (red) or down-regulation (green) of the respective proteins. The characteristics of the differentially expressed proteins in both conditions by IPA analysis prediction were classified in 5 networks and reported in Supplementary Figure S3 and S4. **C.** Protein abundance in HepG2 and **D.** HuH-7cell lines. GAPDH was used as a loading control. Signal intensity values (mean ± S.D.) for RANBP1, RAN, p-NDRG1/NDRG1 and p-MDM2/MDM2, were obtained via densitometric scanning of gels from three independent experiments in treated and untreated HepG2 and HuH-7 cells and are expressed as the percentage of the control and shown under each lane in the blots.

After automatic spot detection, background subtraction and volume normalization, we detected about 650 protein spots in HuH-7 cells treated with SI113 and 620 protein spots in vehicle-treated HuH-7cells. The groups were thus compared, and 87 spots of interest (matches) were used for tandem mass spectrometry analysis. Only reproducibly detected spots were subjected to statistical analysis. A list of significant up- or down-regulated proteins is provided (Supplementary Figure S6). Among differentially expressed proteins, RAN-specific GTPase-activating protein (spot #22) and GTP-binding nuclear protein RAN (spot #76) appeared to be down-regulated following SI113-dependent inhibition of SGK1 (Figure 4, panel A), mimicking what was previously detected upon *SGK1* specific RNA silencing [21].

### Pathway analysis

The software IPA (Ingenuity^®^ Systems, http://www.ingenuity.com) was used to evaluate significant pathways and networks associated with the differentially expressed proteins. We first explored the network characteristics of the differentially expressed proteins in treated HCC cells relative to their controls. IPA analysis predicted five networks (Supplementary Figure S7) of interacting protein clusters according to the identifiers’ HomoloGene to the ortholog information in the Ingenuity Knowledge Base (IKB). Network 1, with a score of 63, comprised 27 nodes that had functions associated with carbohydrate metabolism, energy production, nucleic acid metabolism (Supplementary Figure S8). Network 2 had functions associated with amino acid metabolism, small molecule biochemistry, cellular growth and proliferation (Figure 4, panel B and Supplementary Figure S10). Next, we explored the statistically enriched Molecular and Cellular Functions (*P* value ≤ 0.05, Fisher's exact test implemented in the IPA, corrected with Benjamini-Hochberg method), and identified functions, including: I) Post-Translational Modification (*P* = 1.49^−6^), II) Protein Synthesis (*P* = 1.49^−6^), III) Amino Acid Metabolism (*P* = 6.94^−6^), IV) Small Molecule Biochemistry (*P* = 6.94^−6^), V) DNA Replication, Recombination, and Repair (*P* = 1.37^−5^). Last, we searched for links to diseases and bio-functions and found that differentially expressed proteins were connected with: I) Cancer (n molecules = 67, *P* = 3.19^−2^), II) Cellular Growth and Proliferation (*n* molecules = 35, *P* = 6.56^−5^), III) Cell Death and Survival (*n* molecules = 30, *P* = 1.10E-03), respectively.

### SI113 and protein expression in HepG2 and HuH-7 cells

We also analyzed the expression of proteins that are SGK1 targets and/or substrates by mono-dimensional SDS–polyacrylamide gel electrophoresis followed by Western blot.

After treatment of HuH-7 and HepG2 cells with SI113 for 72 h, the expression of RAN, RANBP1, p-NDRG1, NDRG1, pMDM2 and MDM2 was examined (Figure 4, panels C and D). In treated samples, expression of RAN and RANBP1 as well as phosphorylation of p-MDM2 (serine 166) were reduced, consistent with previously published observations [20, 24]. Phosphorylation of pNDRG1 was clearly reduced in HuH-7 cells (Figure 4, panel D), whereas in HepG2 cells it paralleled the reduction in NDRG1 total abundance (Figure 4, panel C).

### SI113 treatment produced a consistent tumor suppression activity in HCC xenografts in immunocompromised mice

**Figure 5:**
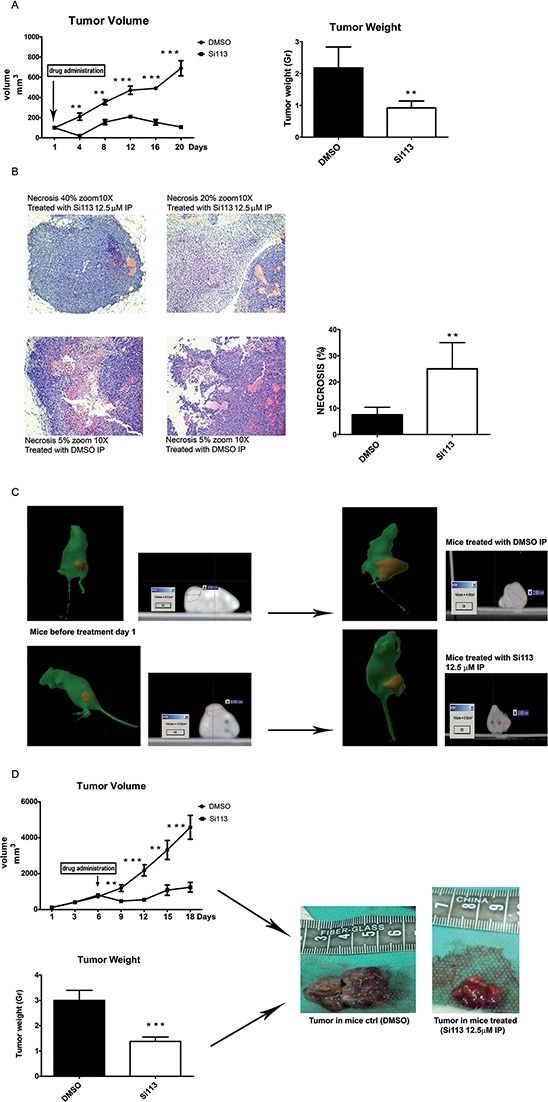
SI113 tumor suppressive activity in HCC xenograft models **A.** NOD/SCID female mice bearing HCC tumors were either untreated or treated with SI113; tumor volumes were measured as indicated in the text. Points, mean; bars, S.D (left panel). On the right panel, histograms represent tumor weights from mice treated with intraperitoneal administration of either SI113 (8 mg/kg/day) or vehicle alone (mean from 5 mice per group). **B.** Microscopic images (10×) of tumor sections (left panel) stained with conventional H & E. Histograms show the extent of necrosis, represented as the mean of 5 mice per group, after treatment with intraperitoneal administration of either SI113 (8 mg/kg/day) or vehicle alone (right panel). **C.** Mice bearing ectopic HCC xenografts (depicted in red) underwent Computed Tomography before (left panel) and after (right panel) intraperitoneal administration of either SI113 (8 mg/kg/day) or vehicle alone. Representative images are shown. **D.** NOD/SCID female mice bearing HCC tumors. Tumor volumes measured as indicated in the text. Points, mean; bars, S.D (upper panel). The histogram represents mean tumor weights (10 mice per group after intraperitoneal SI113 administration (8 mg/kg/day) or vehicle alone (bottom panel). Representative images of xenograft specimens are shown (right panel).*P ≤ 0.05; **P ≤ 0.01; ***P ≤ 0.001.

HuH-7 cells (2.5 × 10^6^) were implanted in the flanks of NOD/SCID female mice for *in vivo* treatment with SI113. We carried out two sets of experiments. In the first one, 10 mice were used, randomly subdivided into two batches. Treatment with SI113 started when tumor volume reached 100 mm^3^, as assessed by manual caliper measurement. The drug (or vehicle) was administered daily and tumor growth was monitored every 4 days. Mice were sacrificed when at least one tumor in either of the two groups reached the volume of 700 mm^3^. Tumors were then excised and weighed, while samples were prepared for histology. An impressive reduction in tumor growth was recorded in the treated batch compared with controls (Figure 5, panel A, left). The difference was already significant on the fourth day after the treatment start and remained significant throughout the duration of the experiment. On day 20, mice in the control group reached the pre-set volume end point (688.57 ± 75.4 mm^3^), whereas mice in the treated group showed a remarkably smaller tumor volume (108.48 ± 22.9 mm^3^, *P* = 0.0009). Measurements of the tumor weights (2.17 ± 0.29 g in controls and 0.75 ± 0.10 g in the treated group, *P* = 0.007) further confirmed the effectiveness of the treatment (Figure 5 panel A, right). In tumor samples analyzed by H&E staining (Figure 5, panel B), necrotic and dead cells were significantly less represented in samples from control compared with treated animals (*P* = 0.01). Data automatically analyzed for staining intensity and specificity are shown in graphic form (Figure 5, panel B, right).

In a second experimental set, 20 mice were enrolled and randomly divided into two batches. HuH-7 cells (3.5 × 10^6^) were implanted in the flank of female NOD/SCID mice as above, but tumors were allowed to grow for 6 days before starting the treatment. CT scanning was performed immediately before and on the last day of treatment, before sacrifice (Figure 5, panel C). At treatment onset, the mean tumor volume was 761 ± 40.4 mm^3^. Mice were randomly assigned to either treatment or control groups, as before. SI113 or vehicle were administered for 12 days and tumor volumes were recorded every 3 days, until at least one tumor in either of the two groups reached a volume of 4500 mm^3^ (Figure 5, panel D, top/left).

Tumor volumes in the treated group were already significantly smaller than in the control group on the third day of treatment and remained significantly smaller until the mice were sacrificed. Weights of the excised tumors (Figure 5, panel D, bottom/left) confirmed that mean value of the tumors from the treated group was significantly smaller than that from the control group (1.38 ± 0.17 g vs. 3 ± 0.39 g, respectively; *P* = 0.001). Representative images of the two tumor groups are provided in Figure 5, panel D, right). No side effects, e.g. weight loss, diarrhea, dermatitis, ulceration, or signs of liver failure were noted in SI113 treated mice. Differences in tumor volumes at various times are detailed in the Figure.

### Synergy of SI113 with radiation-induced growth inhibition and cell death indicates a possible role of SGK1 in radio-resistance

The data so far indicate a detrimental effect of SI113 on HCC progression. We therefore explored the possibility that SI113-dependent inhibition of SGK1 might synergize with radiation therapy in HCC treatment. Curves of sensitivity to ionizing radiations for HepG2 and HuH-7 cells demonstrated a dose-dependent effect on cell viability (details in Supplementary Figure S9).

**Figure 6:**
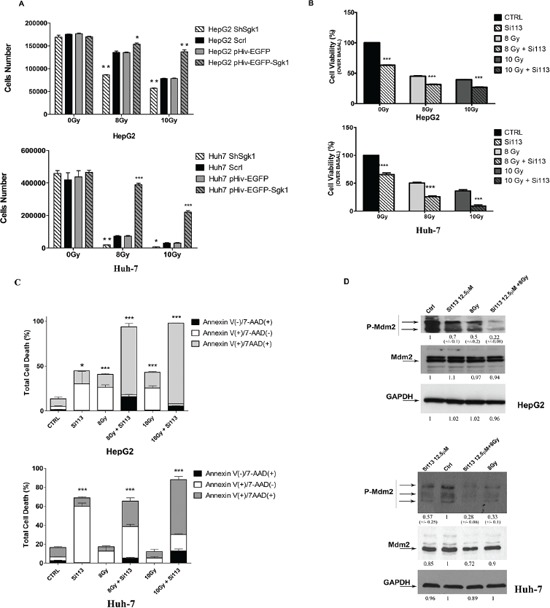
SGK1 inhibition influences cellular radiosensitivity: SI113 synergizes with radiotherapy in HepG2 and HuH-7 cells **A.** ShSGK1 HepG2 and HuH-7 cells, pHIV-EGFP-*SGK1* HepG2 and HuH-7cells were treated with and without radiation, as indicated, at different doses. Histograms represent the number of viable cells in each condition. **B.** HepG2 (top) and HuH-7 (bottom) cell lines. Histograms represent cell numbers after treatment with SI113 (12.5 μM, 72 h) in the absence or presence of radiation (8 or 10 Gy). Results are expressed as the percentage of the number of control cells (HepG2 0 Gy 126000 ± 3058; HuH-7 0 Gy 147666 ± 1454) treated with vehicle alone, and represent the mean ± S.E. of six independent experiments for each cell line. **C.** HepG2 (top) and HuH-7 (bottom) cell lines were treated with SI113 (12.5 μM) or vehicle alone for 72 h in the presence or absence of radiation (8 or 10 Gy). Percentages of cells stained with either Annexin V or 7-ADD (calculated using the Guava Annexin V assay) are shown in the graphs. Histograms depict the percentage of early apoptotic (Annexin V(+)/7-AAD(−)), late apoptotc (Annexin V(+)/7−AAD(+)) and necrotic/dead (Annexin V(−)/7-AAD(+)) cells, after treatment with SI113 and/or radiation. **D.** Immunoblot of P-MDM2/MDM2 72 h after single or combination treatments. As a control, GAPDH was determined in the same blots using an anti-GAPDH antibody in both HepG2 (top) and HuH-7 (bottom) cell extracts.

To verify whether the level of SGK1 expression affected radiosensitivity of liver cancer cell lines, we used lentiviral vectors to produce stably transduced HepG2 and HuH-7 cell lines expressing either *SGK1* specific ShRNA (Sh*SGK1* cells) or wild type *SGK1* (p-Hiv-EGFP-*SGK1* cells) to induce SGK1 silencing or over expression, respectively. After irradiation, cell viability in these cell lines was compared to that of Sh*SCRL* cells and p-HIV-*EGFP* lines, respectively. With both 8 and 10 Gy doses, *SGK1* silencing determined a significant reduction in the number of viable cells, whereas *SGK1* overexpression determined a protective effect, denoted as a significant increase in the number of viable cells, compared with control cells (Figure 6, panel A). The actual differences in cell number between engineered cells lines and their corresponding controls, together with the level of significance, are detailed in Figure Legend and in Supplementary File S2.

We then evaluated the effect of SI113 on radiosensitivity in HepG2 and HuH-7 cells 24 h after plating. Cells were exposed to no radiation (0 Gy), 8 Gy or 10 Gy, with or without treatment with SI113 (72 h), and assayed for cell viability. In both cell lines, SI113 or radiation, as single agents, significantly reduced the number of viable cells, as expected. The combination of SI113 and radiation reduced the number of viable cells more than either agent alone (Figure 6, panel B).

We also examined the effects of SI113 and radiation on the induction of apoptosis in both cell lines using the Guava Nexin assay, as described above. In both cell lines, treatment with either SI113 or radiation significantly increased apoptosis. Treatment with both agents together determined a more pronounced response than either agent alone, reaching 93.5% in cells treated with SI113 plus 10 Gy radiation (Figure 6, panel C). Slightly different results were obtained in HuH-7 cells, as total cell death (early apoptosis, late apoptosis and necrosis) recorded with single treatments increased significantly only when SI113 was added. The combined treatment also dramatically increased the percentage of total dead cells in this cell line, suggesting that the combination determined progression through advanced phases of apoptosis and caused the death of those cells that survived radiation administration protocols (Figure 6, panel C). The actual differences in the percentage of cells in the different phases of apoptosis, together with the level of significance are detailed in Figure Legend, and the graphs listed in Supplementary Figure S10.

MDM2 phosphorylation experiments provided mechanistic insight into the apoptotic process triggered by treatment with SI113 or radiation. SGK1 stabilizes MDM2 via phosphorylation on serine 166, thereby facilitating p53-dependent death [20]. Consistently, we found that treatment of both HepG2 and HuH-7 cells, with either SI113 (72 h) or 8 Gy radiation, significantly reduced MDM2 phosphorylation on serine 166. The combined treatment with SI113 and 8 Gy radiation induced a further reduction in serine 166 phosphorylation, suggesting that radiation synergizes with SGK1 inhibition in reducing MDM2 phosphorylation and stability (Figure 6, panel D).

## DISCUSSION

SGK1 has been recently attributed a role in neoplastic transformation [11–14], and SGK1 specific inhibitors have been tested in several neoplastic models, including colon carcinoma [33–35]. In HCC, SGK1 expression is particularly relevant in highly malignant tumors characterized by epithelial-to-mesenchimal transition (EMT) that supports the underlying liver cirrhosis [17]. A new SGK1 inhibitor [31] has now been tested in two well-established cellular models of HCC, i.e. HepG2 and HuH-7.

We show that SI113-dependent SGK1 inhibition recapitulates the effects of *SGK1*-specific silencing on cell cycle [20, 21]. In both cell lines, SI113 treatment reduced the fraction of cycling cells transiting through G2/M phase and induced a concomitant increase in cell death, consistent with the ability of SGK1 to regulate transcription of RANBP1 [21], a key regulator of the mitotic machinery [36–40]. We also report the identification of 85 proteins differentially modulated by SGK1, some of which are related to RAN and RANBP1 (Supplementary Figure S6). SGK1 inhibition results in down-regulation of RHOA [41, 42], PAK2 [43]and PHB [44], all of which are involved in progression through G2/M. These proteins are also over-expressed in conditions associated with liver metastasis, mitotic instability and drug resistance [42–44]. On the other hand, ST-13 [45, 46] and MLC [47], two down-regulated proteins in HCC, appear to be over-expressed upon SI113-dependent SGK1 inhibition.

Our previous work suggests that SI113 inhibits SGK1 [31, 32]. In the present paper, we demonstrate that the expression of SGK1 is mandatory for SI113 to decrease HCC cell viability and trigger apoptosis; these effects are instead not detected in Sh*SGK1* cells. In these cells, in fact, SI113 had no pro-apoptotic effect, whereas a modest decrease in late apoptotic cells was recorded compared with vehicle-treated SGK1 silenced cells. These apparently incongruous results clearly indicate that SGK1 expression is necessary for SI113 to elicit its pro-apoptotic effect. On the other hand, however, it is possible that, in the absence of SGK1, other unknown SI113 molecular targets exert some effect. This possibility must be taken into account in case of tumors that are insensitive to SI113 or do not express a functional SGK1. SGK1 pharmacological inhibition also recapitulates the effects of S*GK1* specific silencing, on p53 degradation, enhancing the apoptotic response to cellular stresses. Post-translational modifications of p53, which may be important in HCC regulation of cell metabolism in non-transformed hepatocytes [48], are probably crucial in regulating cell proliferation [49, 50]. Both RANBP1/RAN and MDM2/p53 pathways are attributed a central role in cell transformation, in regulation of cell proliferation and in response to mitotic errors. Since SGK1 acts as a critical regulator in both these pathways, we consider that SGK1 overexpression may also have a central role in cancer.

The expression level of MDM2 affects cell radiosensitivity, where decreased levels of MDM2 sensitize cells to ionizing radiations [51], especially in tumors expressing wild-type p53. It is therefore conceivable that the level of expression of SGK1 also affects radiosensitivity. Indeed, we found that *SGK1* gene silencing yielded a clear-cut increase in radiosensitivity, whereas its overexpression induced radioresistance. Moreover, SI113-dependent inhibition of SGK1 synergized with radiotherapy in reducing the fraction of viable cells and causing necro/apoptosis. Interestingly, the combined radiation/SI113 therapy induced an almost complete dephosphorylation of MDM2 on serine 166. We also demonstrate that SI113 treatment inhibits NDRG1 phosphorylation in HuH-7 cell lines and yields a decrease in NDRG1 overall abundance in HepG2 cells. This finding may add another possible mechanism through which SGK1 inhibition might be beneficial in hepatocarcinoma treatment; indeed, NDRG1 has been proposed as a potential therapeutic target for HCC [29].

We finally present *in vivo* assays to test the effect of SI113 in human hepatocarcinoma cells xenografted in immunodeficient mice. The analysis of tumor volume and weight demonstrates that SI113 arrests tumor growth, whereas histology demonstrates high levels of necrosis in tumors from treated animals. No signs of toxicity were observed by histological examination of the livers from S113-treated mice, nor did the mice show signs of generally adverse side effects.

In conclusion, SI113, alone or in synergy with radiotherapy, arrests tumor growth and induces apoptosis and necrosis. These effects are specifically related to SGK1 inhibition, which ultimately turns off several pathways (e.g. RAN/RANBP1, MDM2/p53, NDRG1) that represent convergent targets of the oncogenic function of SGK1.

These results have potential relevance to possible applications in human cancer therapy. In recent years, several drugs have been developed to target the mTOR/AKT1 pathways [52]. These drugs appear to be less effective in tumors characterized by high levels of SGK1 expression [23], moreover they are endowed with intrinsic toxicity, as expected on the basis of the phenotypes observed in mTOR and/or AKT1 knock-out murine models [53, 54]. In contrast, *SGK1* knock-out mice display very subtle phenotypes, elicited in the presence of particular metabolic conditions or cellular stresses [19, 55]. Although more exhaustive toxicity studies of SI113 are needed, from a theoretical point of view, SGK1 inhibition is predicted to be quite safe in normal cells, yet is expected to be powerful in inhibiting proliferation and survival in cancer cells.

## MATERIALS AND METHODS

### HCC cell lines

Human HCC cell lines HuH-7 and HepG2 were obtained from ATCC (Georgetown University in Washington, DC) and cultured as indicated in Supplementary File S1.

### Recombinant DNAs

pHIV-EGFP-SGK1 for SGK1 expression and pLKO.1-puro-ShSGK1 for RNA interference experiments were prepared as indicated in Supplementary File S1 and used to generate lentiviral particles in HEK293T packaging cells. Details for the preparation of virus particles are provided in Supplementary File S1. For negative controls, viral particles were produced in HEK293T cells using either ShScrl (Sigma SHC002V) or pHIV-EGFP, as indicated.

### Infection of cell lines

#### ShSGK1-HuH-7/HepG2 cells

Supernatants from Sh*SGK1*-HEK293T cells were collected and used for transduction of HepG2 and HuH-7 cells in the presence of 8 μg/mL polybrene (Sigma) and selected in the presence of puromycin (1.2 μg/mL). Transduced cells (ShSGK1-HuH-7/HepG2 cells) were compared with control cells (ShScrlHuH-7/HepG2 cells).

#### pHIV-EGFP-SGK1 HuH-7/HepG2 cells

Supernatants from pHIV-EGFP-SGK1-HEK293T cells were collected and used for transduction of HepG2 and HuH-7cells in the presence of 8 μg/mL polybrene (Sigma). Transduced cells (pHIV-EGFP-SGK1HuH-7/HepG2 cells) were compared with control cells (pHIV-EGFP HuH-7/HepG2 cells).

### SI113 treatment

SI113 was developed as previously reported [32]. The drug was diluted (10 mM) in dimethyl sulfoxide (DMSO) and stored at −20°C for *in vitro* studies. For *in vivo* experiments, SI113 (50 mM in DMSO) was diluted 1:5 with saline solution to bring the final concentration to 10 mM. Forty-three μl of this solution were injected by intraperitoneal administration in xenografted NOD/SCID female mice, to obtain a final *in vivo* concentration of approximately 12.5 μM corresponding to 8 mg/kg/day, assuming a volume of drug distribution into the animal of 35 ml.

### Proliferation assays

Cell proliferation was evaluated by measuring the total number of cells with a Burker chamber. Unless otherwise specified, HCC cells were plated at a density of 2 × 10^5^ cells/ml in six-well plates. Viability assays by trypan blue exclusion dye was performed on adherent cells incubated in the absence or presence of SI113, as indicated. Viable cells at each time point were expressed as the percentage of viable cells compared with the vehicle-treated controls.

### Mouse xenograft model and tumor growth delay experiments

Female NOD/SCID mice (4-week-old, Harlan, Indianapolis, IN) were maintained under pathogen-free conditions and given food/water *ad libitum*. Experiments were carried out in accordance with the Catanzaro University Institutional Animal Care and Use Committee guidelines, using an approved protocol. At 6 weeks of age, mice were subcutaneously injected either with 2.5 × 10^6^ or 3.5 × 10^6^ HuH-7 cells, depending on specific experimental design, suspended in 200 μl of a 1:1 solution containing DMEM without serum and Matrigel solution (BD Collaborative Research), in the dorsal posterior-lateral right region. Tumors were allowed to grow for different times in separate experiments, as indicated in the Results section. Mice were randomly assigned to 2 groups of five or ten animals each and administered with either SI113 (treated group) or vehicle alone (DMSO, control group) for five days/week. Tumors were measured every 3 or 4 days by caliper in two perpendicular diameters (a = smaller diameter; b = larger diameter) and the tumor volume was calculated in accordance with the formula: V = π/6 × a2 × b. Mice were subjected to CT scan (Toshiba CT System, Model TSX-021B) after anesthetic induction, before and after the treatment. Mice, under general anesthesia, were sacrificed by vertebral dislocation.

### Immunoblot analysis

SGK1 kinase activity on MDM2 and NDRG1 was evaluated by Western blotting using phospho-specific antibodies, according to previously published methods [20, 22]. The inhibitory effect of SI113 on MDM2 and NDRG1 phosphorylation was monitored as detailed in Supplementary File S1.

### Proteomic analysis, nanoscale LC-MS/MS analysis and pathway analysis

Proteomic analysis and pathway analysis were performed according to previously published methods [56–58], see Supplementary File S1 for a detailed description.

### Cell cycle analysis

Propidium iodide staining was used to evaluate cell cycle effects of SI113 alone or in combination with radiation. HuH-7 and HepG2 cells were treated with SI113 as indicated. Cells, fixed in 70% ethanol, were incubated for 1 h at room temperature in PBS containing propidium iodide (20 μg/ml), 0.1% NP40 and 40 μg/ml ribonuclease, and analyzed by fluorescence activated cell sorter (FACS) analysis using a FACScan© cytometer (Becton Dickinson) using CellQuest software according to previously published methods.

### Apoptosis assay

Guava Nexin Assay was performed as previously published [31]. Four populations of cells can be distinguished in this assay: non-apoptotic cells Annexin V(−) and 7-AAD(−); early apoptotic cells Annexin V(+) and 7-AAD(−); late stage apoptotic cells Annexin V(+) and 7AAD(+); necrotic/dead cells Annexin V(+) and 7AAD(−). The results include counts and percentages of cells in each of the quadrant-defined populations, as well as the mean fluorescence intensity of Annexin V and 7-AAD for each population.

Multi caspase assay was conducted using the Guava Caspase Kit (Millipore 4500-0500) following the manufacturer's instructions. Briefly, cells were stained with the Guava Caspase 7 AAD reagent (Millipore 4000-0290) in the presence of 7AAD as an indicator of cell membrane integrity. Four populations of cells can be distinguished in this assay : Lower-left quadrant: viable cells [Caspase Reagent(−) and 7-AAD(−)]; Lower-right quadrant: cells in the middle stages of apoptosis [Caspase Reagent(+) and 7-AAD(−)]; Upper-right quadrant: cells in the late stages of apoptotic or dead [Caspase Reagent(+) and 7-AAD(+)]; Upper-left quadrant: necrotic cells [Caspase Reagent(−) and 7-AAD(+)]. More details are given in Supplementary File S1.

### Radiation therapy

Cells were plated in 100-mm Ø tissue culture dishes, allowed to attach for 24 h, and treated with two different doses of radiation (8 and 10 Gy) at room temperature (1.8 Gy/min, 98 cm Source Surface Distance (SSD) by using a 6 MV photon linear accelerator (CLINAC 600 Varian).

### Hematoxylin and eosin staining

Nine paraffin-embedded tissue blocks of human hepatocellular carcinoma xenografts were prepared. Sections were stained with conventional hematoxylin and eosin (H & E) and analyzed under a Nikon ECLIPSE 55i microscope with digital camera (HESP Technology).

### Statistical analysis

Tests were done in triplicate and experiments performed at least three times. Results are expressed as a mean ± Standard Error (S.E.) or Standard Deviation (S.D.). Differences between groups were analyzed using the Student's two-tailed *t* test (GraphPad Prism v5). Asterisks denote statistical significance as indicated in the legends.

## SUPPLEMENTARY FIGURES


