# Supplementary material for: Preclinical model in HCC: the SGK1 kinase inhibitor SI113 blocks tumor progression in vitro and in vivo and synergizes with radiotherapy
Source: Oncotarget. 2015 Oct 8;6(35):37511–25. doi: 10.18632/oncotarget.5527 (PMC4741945; doi:10.18632/oncotarget.5527)
Supplement: Supplementary file 1 [file oncotarget-06-37511-s001.pdf]

SUPPLEMENTARY FILE S1 (METHODS)

HCC cell coltures

Human HCC cell lines HuH-7 and HepG2 were cultured in DMEM (Life Technologies, Inc., Grand Island, NY) supplemented with 10% fetal bovine serum and 1% penicillin-streptomycin solution (Aurogene) and in RPMI (Life Technologies, Inc., Grand Island, NY) supplemented with 10% fetal bovine serum, 1% penicillin-streptomycin solution, 1% MEM-Eagle Non-essential amino acids solution (Biological Industries) and 1% L-Glutamine (Invitrogen-Gibco) respectively. Cells were cultured at 37°C in a humidified atmosphere of 5%CO2 and 95% air.

Recombinant DNAs

Preparation of pHIV-EGFP expression vector for SGK1 expression, pHIV-EGFP-SGK1

The coding sequences of wild-type *SGK1* within PcDNA4-TO (empty vector) was amplified by PCRusing specific primers flanked by restriction sites necessary for cloning into the XbaI and BamHI, sites of the pHIV-EGFP expression vector (Addegene plasmid 21373) to obtain a self-inactivating lentiviral plasmid for co-expression of *SGK1* and EGFP (CGT TCT AGA GCG GTG GTG ATGACG GTG AAA ACT GAG) and (ATG GGA TCC TCA GAG GAA AGA GTC CGT G).

Vectors used for RNA interference experiments, pLKO.1-puro-ShSGK1

For RNA interference experiments we used the human *SGK1* MISSION shRNA set (fivesingle hairpins individually cloned into pLKO.1-puro; Sigma) (*SGK1* sequence NM_005627 GenBank). Scrambled RNA; Sigma SHC002V was used as a negative control.

Preparation of virus particles

p-HIV-EGFP-*SGK1* and pLKO.1-puro-Sh*SGK1* were used to generate lentiviral particles in HEK293T packaging cells. Subconfluent HEK293T cells were co-transfected with 8 µg *SGK1*MISSION shRNA set or 8 µg pHIV-EGFP and pHIV-*SGK1*, 10 µg pCMV-delta8.9, and 2 µg pCMV-VSVG per 100-mm tissue culture plate by Lipofectamine™ 2000. Viral particles to be used in HepG2 were produced in HEK293T cells grown in RPMI1640, Viral particles to be used in HuH-7 were produced in HEK293T cells grown in DMEM. For negative controls viral particles were produced in HEK293T cells using either Sh*Scrl* (Sigma SHC002V) or pHIV-*EGFP* as indicated.

Immunoblot analysis

SI113-dependent inhibition of SGK1 kinase activity was monitored on phosphorylation of endogenous substrates (MDM2 and NDRG1) by Western blotting. To analyze the inhibition of SGK1 by Si113, HCC cells were cultured in the presence of 12.5 µM SI113 for 72 h. Cells were then lysed[lysis buffer: 50 mM Tris-HCl, pH 7.4, 0.15 M NaCl, 0.5% IGEPAL, 25 mM NaF, 1 mM DTT, 1 mM Na_3VO_4 plus protease inhibitor cocktail 10 × (Sigma-Aldrich, St. Louis, MO)], extracts were separated by 12% SDS-polyacrylamide gel electrophoresis and blotted on nitrocellulose membrane 0.45 µm (Bio-Rad, Hercules, CA). Blots were blocked overnight (T-TBS + 5% non-fat dry milk) and probed with either anti-phospho-serine 166 MDM2 rabbit polyclonal antibody (#35215 Cell Signaling, Danvers, MA) or anti Phospho–NDRG1 Rabbit monoclonal (Thr346) (D98G11) (#5482 Cell Signaling Danvers, MA) at a 1:1000 dilution in T-TBS 1X containing 3% BSA or 5% non-fat dry milk. Total MDM2 and total NDRG1 were detected by MDM2 rabbit immunoglobulins (sc-813 Santa Cruz Biotechnology, Inc. Santa Cruz, CA) and NDRG1 rabbit immunoglobulins (#9485 Cell Signaling Danvers, MA), respectively. Bands were visualized by chemiluminescence using the ECL Kit (GE Healthcare). Sample loading controls (10–30 µg/well) were obtained by membrane stripping followed by immunostaining with antibodies against GAPDH (sc-25778. Santa Cruz Biotechnology, Inc. Santa Cruz, CA). In order to substantiate the synergic effect between SI113 and radiotherapy, after both treatments, as explained in the radiotherapy section of Material and Methods, protein samples in every condition were probed with antibodies against P-MDM2 and total MDM2. To investigate the effects on RANBP1 and RAN expression, protein extract from treated or untreated HepG2 and HuH-7 cells lines, prepared and loaded as above, were probed with anti-RANBP1 (goat polyclonal sc-1160 Santa Cruz Biotechnology, Inc. Santa Cruz, CA) and anti-RAN (goat polyclonal sc-1156 Santa Cruz Biotechnology, Inc. Santa Cruz, CA).

Proteomic analysis

Cell lines were washed with PBS(1X) and lysed at 0°C for 30 min in lysis buffer (120 mM NaCl, 30 mM KCl, 0.1% DTT, 0.5% Triton X-100) supplemented with protease and phosphatase inhibitor cocktail (Halt Protease Inhibitor Cocktail/ Halt Phosphatase Inhibitor Cocktail, Thermo Fisher Scientific Inc.). Cell lysate was sonicated at 4°C for 10 sec and subsequently centrifuged at 15000 × g for 20 min. Supernatant was carefully removed and

protein content was measured by the Bradford method (Bio-Rad); supernatants were stored at 80°C. 500 µg of protein was diluted into Isoelectrofocusing (IEF) sample buffer containing 8 M urea, 4% CHAPS, 0.1 M DTT, 0.8% pH 3–10 nonlinear (NL) carrier ampholyte buffer. IEF was carried out on non-linear immobilized pH gradients (pH 3–10 NL; 24-cm-long IPG strips; GE Healthcare). The first dimension IPG strips were run on a GE Healthcare IPGph or unit, until a total of 70,000 Vh was reached. Prior to SDS-PAGE, IPG strips were equilibrated with a dithiothreitol (10 mg/mL) SDS equilibration solution followed by treatment with iodoacetamide (25 mg/mL) SDS equilibration solution as described in the GE Healthcare Ettan DIGE protocol. Second dimension separation was run on 10% SDS-polyacrylamide gels, (2 W/gel; 25°C) until the bromophenol blue dye front reached the end of the gels. Gels were stained with MS-compatible silver staining procedure. Protein spots were analyzed in terms of volume performed using the Image Master 2D-Platinum software, version 6.0 (GE Healthcare BioSciences). Spot digestion was performed as previously reported. Briefly, electrophoretic spots, obtained from analytic 2D gels, were manually excised, destained, and acetonitrile-dehydrated. They were then rehydrated in trypsin solution, and in-gel protein digestion was performed by overnight incubation at 37°C. The resulting trypticpeptides were purified byPierce® C18 Spin Columns (Thermo Fisher Scientific Inc.) according to the manufacturer's procedure, eluted with 40 µL of 70% acetonitrile and dehydrated in a vacuum evaporator. Each purified tryptic peptide was analyzed through Nanoscale LC-MS/MS.

Nanoscale LC-MS/MS analysis

LC-MS/MS analysis was performed using an Easy LC 1000 nanoscale liquid chromatography (nano LC) system (Thermo Fisher Scientific, Odense, Denmark). The analytical nano LC column was a pulled fused silica capillary, 75 µm i.d., in-house packed to a length of 10 cm with 3 µm C18 silica particles from Dr.Maisch (Entringen, Germany). The peptide mixtures were loaded at 500 nL/min directly onto the analytical column. A binary gradient was used for peptide elution. Mobile phase A was 0.1% formic acid, 2% acetonitrile, whereas mobile phase B was 0.1% formic acid, 80% acetonitrile. Gradient elution was achieved at 350 nL/min flow rate, and ramped from 0% B to 30% B in 15 minutes, and from 30% B to 100% B in additional 5 minutes; after 5 minutes at 100% B, the column was re-equilibrated at 0% B for 10 minutes before the subsequent injection. MS detection was performed on a quadrupole-orbitrap mass spectrometer Q-Exactive (Thermo Fisher Scientific, Bremen, Germany) operating in positive ion mode, with nanoelectrospray (nESI) potential at 1800 V applied on the column front-end via a tee piece. Data-dependent acquisition was performed by using a top-5 method with resolution (FWHM), AGC target and maximum injection time (ms) for full MS and MS/MS of, respectively, 70,000/17,500, 1e6/5e5, 50/400. Mass window for precursor ion isolation was 2.0 m/z, whereas normalized collision energy was 30. Ion threshold for triggering MS/MS events was 2 and 4. Dynamic exclusion was 15 s. Data were processed using Proteome Discoverer 1.3 (Thermo Fisher Scientific, Bremen, Germany), using Sequest as search engine, and the HUMAN-ref prot-isoforms. Fasta as sequence database. The following search parameters were used: MS tolerance 15 ppm; MS/MS tolerance 0.02 Da; fixed modifications: carbamidomethylation of cysteine; variable modification: oxidation of methionine, phosphorylation of serine, threonine and tyrosine; enzyme trypsin; max. missed cleavages 1; taxonomy Human. Protein hits based on two successful peptide identifications (Xcorr > 2.0 for doubly charged peptides, >2.5 for triply charged peptides, and >3.0 for peptides having a charge state >3) were considered valid.

Pathway analysis

Ingenuity® Pathway analysis (Ingenuity Systems, http://www.ingenuity.com) was performed to examine functional correlations within differentially expressed proteins. IPA constructs hypothetical protein interaction clusters on the basis of a regularly updated Ingenuity Pathways Knowledge Base. Data sets containing protein identifiers and corresponding expression values were uploaded into the application. Differentially expressed proteins were overlaid onto global molecular networks developed from information contained in the knowledge base. Networks were then algorithmically generated based on their connectivity. Networks were "named" on the most common functional group(s) present. Canonical pathway analysis acknowledged function-specific proteins significantly present within the networks.

Apoptosis assay

Guava nexin assay

The Guava Nexin Assay utilizes Annexin V-PE to detect Phosphatidylserine (PS) on the external membrane of apoptotic cells. The cell-impermeant dye, 7-AAD, is also used in the assay as an indicator of cell membrane structural integrity. 7-AAD is excluded from live, healthy cells as well as early apoptotic cells. Four populations of cells can be distinguished in this assay: non-apoptotic cells AnnexinV(−) and 7-AAD(−);

early apoptotic cells Annexin V(+) and 7-AAD(−); late stage apoptotic Annexin V(+) and 7AAD(+); necrotic/dead cells Annexin V(−) and 7AAD(+). Cells, treated as indicated, were prepared for incubation with 1% BSA. 2×10^4 cells were incubated with 100 µl Guava Nexin Reagent (Lot No. 14-0032, Merck Millipore), a pre-made cocktail containing Annexin V-PE and 7-AAD in buffer, in a 200 µl final volume. After 20 min incubation at room temperature in the dark, samples were ready to be acquired on a Guava System. The results include counts and percentages of cells in each of the quadrant-defined populations, as well as the mean fluorescence intensity of Annexin V and 7-AAD for each population.

Caspase assay

To allow direct determination of the cell concentration and percent of live, apoptotic, dead and necrotic populations present in cultures, as distinguished by the presence or absence of activated caspases and/or an intact plasma membrane, Huh7 and HepG2 cell lines were treated as indicated above, and staining with the Guava Caspase Kit (Millipore, 4500-0500). The Kit contains a Guava Caspase reagent (Millipore, 4100-1320), which is an inhibitor consisting of a peptide specific for each caspase active site, conjugated to sulforhodamine (SR) fluorochrome. Once inside the cells, the caspase inhibitor binds covalently to the caspase that have been activated while any unbound reagent can diffuse out of the cell and be washed away. Cells showing significant positive staining correspond to cells in the population committed to the apoptotic pathway. Along with the caspase inhibitor is included 7-AAD as an indicator of membrane structural integrity. 2×10^4 cells/ml were used for the Guava Caspase Assay. Cells are incubated with the Guava Caspase Reagent for 1 hour, washed with Apoptosis Wash buffer (Millipore, 4200-0162), stained with the Guava Caspase 7-AAD Reagent (Millipore, 4000-0290) for 10 minutes, and acquired on Guava System. The results include the count and percentage of cells in each of the quadrant-defined populations, as well as the mean fluorescence intensity of Caspase Reagent and 7-AAD Reagent for each population of cells untreated or treated with SI113 12.5 µM for 72 h. Four populations of cells can be distinguished in this assay: Lower-left quadrant: viable cells [Caspase Reagent(−) and 7-AAD(−)]; Lower-right quadrant: cells in the middle stages of apoptosis [Caspase Reagent(+) and 7-AAD(−)]; Upper-right quadrant: cells in the late stages of apoptotic or dead [Caspase Reagent(+) and 7-AAD(+)]; Upper-left quadrant: necrotic cells [Caspase Reagent(−) and 7-AAD(+)].

SUPPLEMENTARY FILE S2 (RESULTS) QUANTITATIVE DETAILS AND SIGNIFICANCE

Figure 1

SI113, a new inhibitor of SGK1, strongly reducescell viability in HCC cells

Statistical significance: Treated vs. untreated HepG2 cells (48 h Si113 12.5 µM $P = 0.023$; Si113 25 µM $P = 0.0031$; Si113 50 µM $P = 0.0040$. 72 h Si113 12.5 µM $P = 0.0099$; Si113 25 µM $P = 0.0096$; Si113 50 µM $p = 0.0087$). Treated vs. untreated HuH-7cells (48 h Si113 12.5 µM $P = 0.023$; Si113 25 µM $P = 0.0051$; Si113 50 µM $P = 0.0040$. 72 h Si113 12.5 µM $P = 0.013$; Si113 25 µM $P = 0.0035$; Si113 50 µM $P = 0.0008$). *$P \leq 0.05$; **$P \leq 0.01$; ***$P \leq 0.001$.

SI113 inhibits cell cycle progression and induced apoptosis in HuH-7 and HepG2 cell lines in a time-dependent manner (Figure 2A–2D)

n HepG2, a significant reduction of the cell population in the G2/M phase was observed after 72 h of treatment (9.90 ± 0.39% in vehicle-treated, vs. only 0.13 ± 0.13% of the SI113-treated cell population ($P = 2.08^{-5}$). Concomitant with this, SI113 treatment significantly increased the percentage of <G1 hypodiploid cells (1.96 ± 0.12% in vehicle-treated compared with 7.42 ± 1.89% in SI113-treated cells, $P = 0.04$) (Figure 2, panel A). In HuH-7, the effect of SI113 was readily evident already after 48 h: at this time point, the G2-M phase cell population decreased (26.83 ± 0.76% in vehicle-treated cells vs. 20.3 ± 0.70% in SI113-treated cells, $P = 0.003$), with a stronger loss of G2/M phase cells after 72 h of treatment (26.1 ± 0.54% in vehicle-treated vs. 12.67 ± 3.61% in SI113-treated cultures, $P = 0.021$). This was again accompanied by a strong increase in the percentage of <G1 hypodiploid cells in HuH-7 cultures as well (4.32 ± 0.29% in vehicle-treated compared with 17.14%, in SI113-treated cells after 48 h, $P = 0.0008$, and 4.4 ± 0.1% in vehicle-treated compared with 33.3 ± 3.61% in SI113-treated cells after 72 h, $P = 0.0005$) (Figure 2, panel C, Supplementary File S1 and Supplementary File S2).

HepG2 and HuH-7 cells treated with SI113 for 24, 48 and 72 h were also analyzed by cytofluorimetry after dual staining with Annexin V for apoptosis determination, and 7-amino-actinomycin D (7-AAD) to detect necrotic cells with a compromised (permeable) membrane structure. A significant increase in total cell death was demonstrated in SI113-treated cells at each time point in both HepG2 and HuH-7cultures (Figure 2, panel B and D).

In particular, in HepG2 cells (Figure 2, panel B), treatment with SI113 for 24 h yielded a significant increase in early apoptotic cells (26.7 ± 3.79% after treatment and 7.2 ± 0.23% in control cells) In HuH-7cells (Figure 2, panel D); the same treatment yielded increase in necrotic and, particularly significantly, in late apoptotic cells (4.46 ± 0.29% and 1.56 ± 0.12% respectively in the treated population vs. 2.6 ± 0.50% and 0.5 ± 0.2% respectively in control samples). As shown in Figure 2, the induction of apoptosis in SI113-treated samples became gradually more significant in both cell lines after prolonged treatment (compare 72 to 24 hours in panels B and D). In HepG2 cells, SI113 increased the fraction of early apoptotic cells (4.0 ± 0.6% in control vs. 56.7 ± 0.49% in treated samples after 48 h, 6.16 ± 0.20% in control to 52.0 ± 3.08% in treated samples after 72 h). HuH-7 cells (Figure 2, panel D) also proved more prone to necrosis after treatment with SI113 at the same time points. After 48 h, the percentage of late apoptotic cells increased from 1.6 ± 0.2% in control to 5.13 ± 0.3% in treated samples, whereas the percentage of necrotic/dead cells increased from 0.26 ± 0.03% in control to 3.13 ± 0.46% in treated cells. After 72 h, the percentage of late apoptotic cells increased (1.2 ± 1.15% in control to 14.3 ± 0.55% in treated cells) and the percentage of necrotic/dead cells increased from 0.06 ± 0.06% in control to 0.9 ± 0.1% in treated cells. The data indicate therefore that SI113 gradually suppressed the fraction of cycling cells form the treated cell populations and concomitantly activated the apoptotic response pathways in a highly significant manner over time.

Statistical significance compared with vehicle-treated controls: HepG2 cells (24 h Si113 12.5 μM AnnexinV(+)/7-AAD(−) P = 0.006; 48 h Si113 12.5 μM Annexin V(+)/7-AAD(−) P = 3.69 E-07; 72 h Si113 12.5 μM Annexin V(+)/7-AAD(−) P = 0.0001. HuH-7 24 h Si113 12.5 μM AnnexinV(+)/7-AAD(+) P = 0.03, Annexin V(−)/7-AAD(+) p = 0.007. HuH-7cells 48 h Si113 12.5 μM AnnexinV(+)/7-AAD(+)P = 0.0008, Annexin V(−)/7-AAD(+)P = 0.003. 72 h Si113 12.5 μM AnnexinV(+)/7-AAD(+) P = 0.0005, Annexin V(−)/7-AAD(+) P = 0.003. The asterisks indicate the highest degree of significance calculated in each condition. *$P \leq 0.05$; **$P \leq 0.01$; ***$P \leq 0.001$.

Evidences for selectivity of SGK1 targeting by SI113 (Figure 3B and 3C)

SI113 did not affect the viability of SGK1-interfered cultures (dashed right histogram), while significantly affected that of ScrlHuH-7 cells, in which the number of viable cells decreased to 30% upon SI113 treatment (P = 0.008) (Figure 3, panel B).

Stable Scrl or Sh*SGK1*-expressing HuH-7 cells treated with SI113 for 48 h were also analyzed by cytofluorometry after staining with Annexin V and 7 ADD (Figure 3, panel C). The fraction of cells in early apoptosis was higher in Sh*SGK1*-HuH-7 cells (4.46 ± 0.23%) compared with Scrl-HuH-7cells (2.76 ± 0.14%, P = 0.003), consistent with previous observations (22). Treatment for 72 h with SI113 increased all stages of cell death, i.e early apoptosis (2.76 ± 0.14% to 4.53 ± 0.53, P = 0.04), late apoptosis 6.8 ± 0.55% to 14.5 ± 1.97%, P = 0.019; necrotic/dead cells (0.53 ± 0.13% to 1.66 ± 0.14%, P = 0.004 compared with untreated samples), selectively in Scrl-HuH-7.

SI113 had instead no pro-apoptotic effect in Sh*SGK1*-HuH-7cells, in which - if anything - a modest decrease in late apoptotic cells was recorded (7.7 ± 0.3%to 4.5 ± 1%) in cultures treated with SI113 (P = 0.04), compared with vehicle-treated controls. Taken together, these data strongly suggest that SGK1 function is necessary for SI113 to elicit its pro-apoptotic effect.

A possible role of SGK1 in radio-resistance is indicated by SI113 synergy with radiation-induced growth inhibition and cell death (Figure 6A–6C)

The data so far indicate a detrimental effect of SI113 on hepatocarcinoma progression. Because radiotherapy is employed to treat hepatocarcinoma, we sought to evaluate the possibility that SI113-dependent inhibition of SGK1 synergizes with radiation therapy in HCC treatment. We preliminarily determined the curve of sensitivity to ionizing radiations for HepG2 and HuH-7cells. A dose-dependent effect on cell viability was observed for both cell lines (details in Supplementary Figure S6).

At this point, in order to verify whether the level of SGK1 expression affected radiosensitivity of liver cancer cell lines, we used a HIV-based lentiviral vector to produce stably transduced HepG2 and HuH-7 cell lines expressing either *SGK1* specific or wild type to induce SGK1 silencing or over expression, respectively. Cell viability of ShRNA (Sh*SGK1* cells) and *SGK1* (p-Hiv-EGFP-*SGK1)* cells, after exposure to radiation, was compared with that of Sh*SCRL* cells and p-HIV-*EGFP* lines, respectively. In particular, in Sh*SCRL* HepG2 cells (Figure 6, panel A, top), the number of viable cells decreased from 175187 ± 921 to 135667 ± 3170 and 78528 ± 804, upon exposure to 8 Gy and 10 Gy, respectively. In Sh*SGK1* HepG2 cells, the number of viable cells decreased from 169333 ± 4338 to 86333 ± 882 and 57333 ± 667 upon exposure to 8 Gy and 10 Gy, respectively. In pHiv-*EGFP* HepG2 cells, the number of viable cells decreased from 176667 ± 1668 to 135333 ± 1454 and 78533 ± 1074 upon exposure to 8 Gy and 10 Gy, respectively and in pHIV-EGFP SGK1 cells the number of viable cells decreased from 170333 ± 882 to 153817 ± 2476 and 137167 ± 4517, upon exposure to 8 Gy and 10 Gy, respectively. All differences between *SGK1*-

enigeneered cell lines and their relative controls were statistically significant, as indicated in the figure legend.

Similarly in Sh*SCRL*HuH-7cells (Figure 6, panel A, bottom), the number of viable cells decreased from 419333 ± 43817 to 72433 ± 4438 and 29289 ± 3667, upon exposure to 8 Gy and 10 Gy, respectively. In Sh*SGK1*HuH-7 cells, the number of viable cells decreased from 440688 ± 9875 to 19682 ± 183 and 6663 ± 138 upon exposure to 8 Gy and 10 Gy, respectively. In pHiv-EGFP HuH-7 cells, the number of viable cells decreased from 437333 ± 38068 to 72666 ± 4376 and 30000 ± 3858, upon exposure to 8 Gy and 10 Gy, respectively and in p-HIV-EGFP-*SGK1* cells the number of viable cells decreased from 465400 ± 12535 to 389333 ± 8753 and 220666 ± 9272.

Figure 6 A statistical significance : HepG2 8 Gy, Sh*SGK1* vs. ShScrl $P = 0.0004$, pHiv-EGFP-*SGK1* HepG2 vs. pHiv-EGFP HepG2 $P = 0.008$. 10 Gy, ShSGK1 vs. ShScrl $P = 0.006$, pHiv-EGFP-*SGK1* HepG2 vs. pHiv-EGFP HepG2 $P = 0.007$. HuH-**7** 8 Gy, Sh*SGK1* vs. ShScrl $P = 0.001$, pHiv-EGFP *SGK1*HuH-7 vs. pHiv-EGFP HuH-7 $P = 0.01$; 10 Gy, Sh*SGK1* vs. ShScrl $P = 0.01$, pHiv-EGFP *SGK1*HuH-7 vs. pHiv-EGFP HuH-7 $P = 0.001$.

Effect of SI113 on radiosensitivity. We also examined the effects of SI113 and radiation on the induction of apoptosis in both cell lines.

Figure 6 B statistical significance : HepG2 cells. SI113 vs. control $P = 0.0001$; 8 Gy vs. control $P = 3.60^{-5}$; 10 Gy vs. control $P = 1.58^{-5}$; SI113 + 8 Gy vs 8 Gy $P = 0.0004$; SI113 + 8 Gy vs SI113 $P = 2.28^{-6}$; SI113 + 10 Gy vs. 10 Gy $P = 0.0007$; SI113 + 10 Gy vs. Si113 $P = 1.49$ E-05. HuH-7cells. Si113 vs. control cells $p = 0.0003$; 8 Gy vs. control $P = 1.17^{-5}$; 10 Gy vs. control $P = 1.31^{-5}$; SI113 + 8 Gy vs 8 Gy$P = 0.0002$; SI113 + 8 Gy vs SI113 $P = 0.0002$; SI113 + 10 Gy vs. 10 Gy$P = 0.001$; SI113 + 10 Gy vs.SI113 P = 0.0001.

Guava Nexin assay (Figure 6, panel C). In particular, in HepG2 cells, the percentage of total cell death (early apoptosis, late apoptosis and necrosis) increased from 13.4% in control cells to 44.5%, 40.6% and 42.9% in cells treated with either SI113, 8 Gy or 10 Gy radiation, respectively. Interestingly, the treatment with the two agents together determined a dramatic increase in total death, up to 93.5% in cells treated with SI113 and 8 Gy radiation and 97.8% in cells treated with SI113 and 10 Gy radiation, accounting for the almost complete annihilation of malignant cells.

When SI113 and radiation were separately administered as single agents to HepG2 cells, the increase in total cell death was due to an increase in both early apoptotic cells (3.6% in control, compared with 29.7% in SI113-treated and 25.7% in 8 Gy and 24.4% in 10 Gy radiation treated cells) and late apoptotic cells (8.4% in controls vs. 14.3% in SI113 treated,14.3% in 8 Gy and 17.6% in 10 Gy radiation-treated cells). When both SI113 and radiation were used in combination, the percentage of late apoptotic cells grew as high as to 75.5 ± 3.84 (8 Gy) and 90.1 ± 0.25% (10 Gy).

Huh 7 cells gave slightly different results, as total cell death (early apoptosis, late apoptosis and necrosis) recorded with single treatments increased significantly only with SI113 (68.6% compared with 16.26% in controls,17.2% with 8 Gy and 12% with 10 Gy radiation. The increase in total cell death induced by SI113 as a single agent was mainly due to an increase early apoptotic cells (3.8% in control cells, 59.2% in SI113-treated cells).

Combined treatment with both SI113 and radiation, yielded total cell death of 65.3% (SI113 and 8 Gy) and 87.8% (SI113 and 10 Gy radiation).

When radiation was used as a single agent, early apoptosis (3.8% in controls) increased to 12.6% ± 4.24 (8 Gy) and 5.2% ± −1.29 (10 Gy); late apoptosis was 4.5 ± 0.9% in 8 Gy and 6.76 ± 2.35% in 10 Gy treated cells. Interestingly, when radiation and SI113 were used in combination, late apoptotic cells increased to 26.9 ± 3.31% cells (8 Gy) and 57.7 ± 3.3% (10 Gy); concomitant with this, necrosis increased to 5.3 ± 0.6% (8 Gy) and 12.8 ± 2.1% (10 Gy), suggesting that the combination determined progression through advanced phases of apoptosis and caused the death of those cells that survived radiation administration protocols.

Figure 6 C statistical significance : HepG2 cells Si113 vs. control Annexin V(+)/7-AAD(+) $P = 0.03$; Si113 + 8 Gy vs.8 GyAnnexin V(+)/7-AAD(+) $P = 0.0001$; Annexin V(−)/7-AAD(+) $P = 0.004$; SI113 + 8 Gy vs SI113 Annexin V(+)/7-AAD(+) $P = 9.1^{-5}$, Annexin V(−)/7-AAD(+) $P = 0.004$; SI113+ 10 Gy vs. 10 Gy Annexin V(+)/7-AAD(+) $P = 1.1^{-7}$, Annexin V(-)/7-AAD(+) $P = 0.0002$; SI113 + 10 Gy vs. SI113 12.5 µM Annexin V(+)/7-AAD(+) $P = 1.3^{-9}$, Annexin V(−)/7-AAD(+) $P = 0.0001$; 8 Gy vs. control cells Annexin V(+)/7-AAD(−) $P = 0.001$, Annexin V(+)/7-AAD(+) $P = 0.05$; 10 Gy vs. control cells Annexin V(+)/7-AAD(+) $P = 0.01$. HuH-7 cells SI113 vs. control cells Annexin V(+)/7-AAD(−) $P = 0.0001$, Annexin V(−)/7-AAD(+) $P = 0.01$; SI113+ 8 Gy vs. 8 Gy Annexin V(+)/7-AAD(−) $P = 0.01$, Annexin V(+)/7-AAD(+) $P = 0.002$, Annexin V(−)/7-AAD(+) $P = 0.001$; SI113 + 8 Gy vs.SI113 Annexin V(+)/7-AAD(+) $P = 0.007$, Annexin V(−)/7-AAD(+) $P = 0.001$; SI113 + 10 Gy vs. 10 Gy Annexin V(+)/7-AAD(−) $P = 0.001$, Annexin V(+)/7-AAD(+) $P = 0.0002$, Annexin V(−)/7-AAD(+) $P = 0.003$; Si113 + 10 Gy vs Si113 Annexin V(+)/7-AAD(+) $P = 0.0001$, Annexin V(−)/7-AAD(+) $P = 0.004$. The asterisk is always referred to the point of greatest significance as values in the figures legend $*P \leq 0.05$; $**P \leq 0.01$; $***P \leq 0.001$.

SUPPLEMENTARY FIGURES

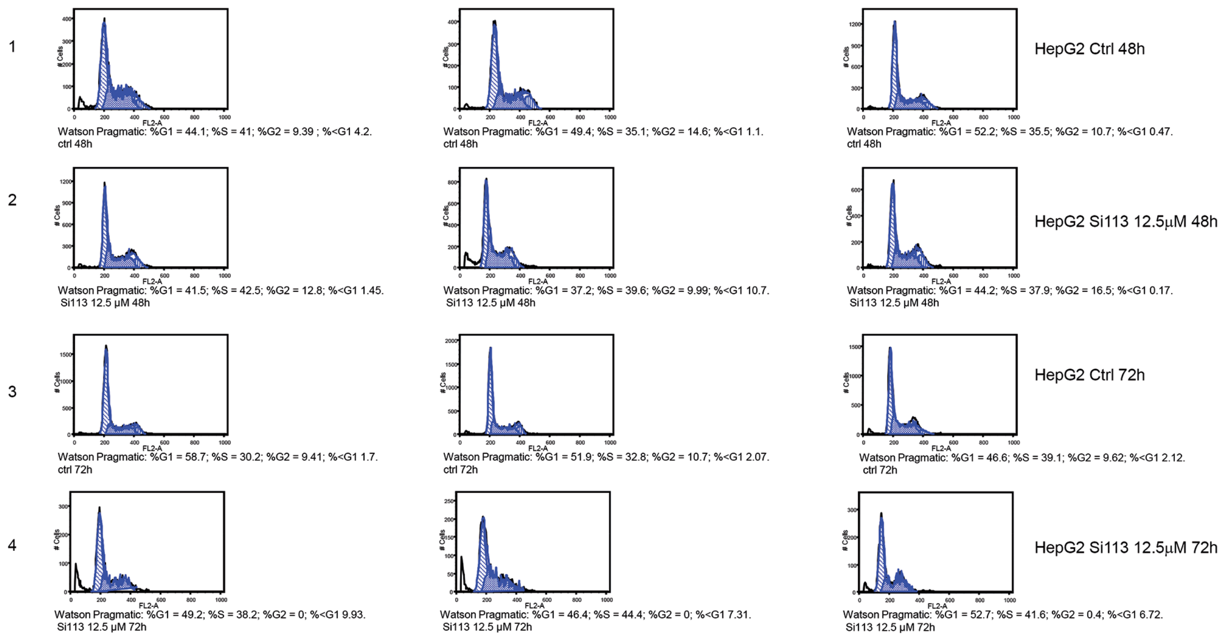

Supplementary Figure S1: Histograms of cell cycle analysis for HepG2 cell line (Figure 2, panel A).

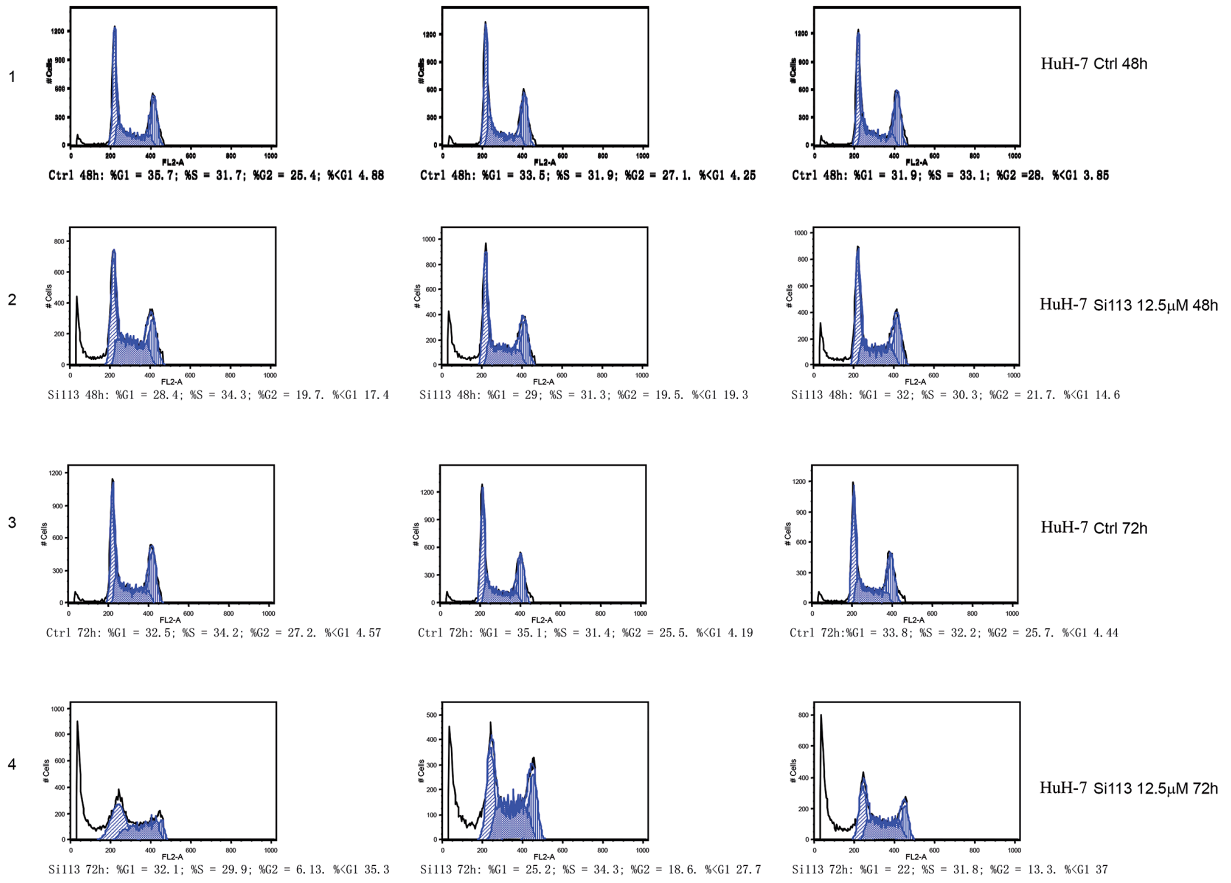

Supplementary Figure S2: Histograms of cell cycle analysis for HuH-7 cell line (Figure 2, panel C).

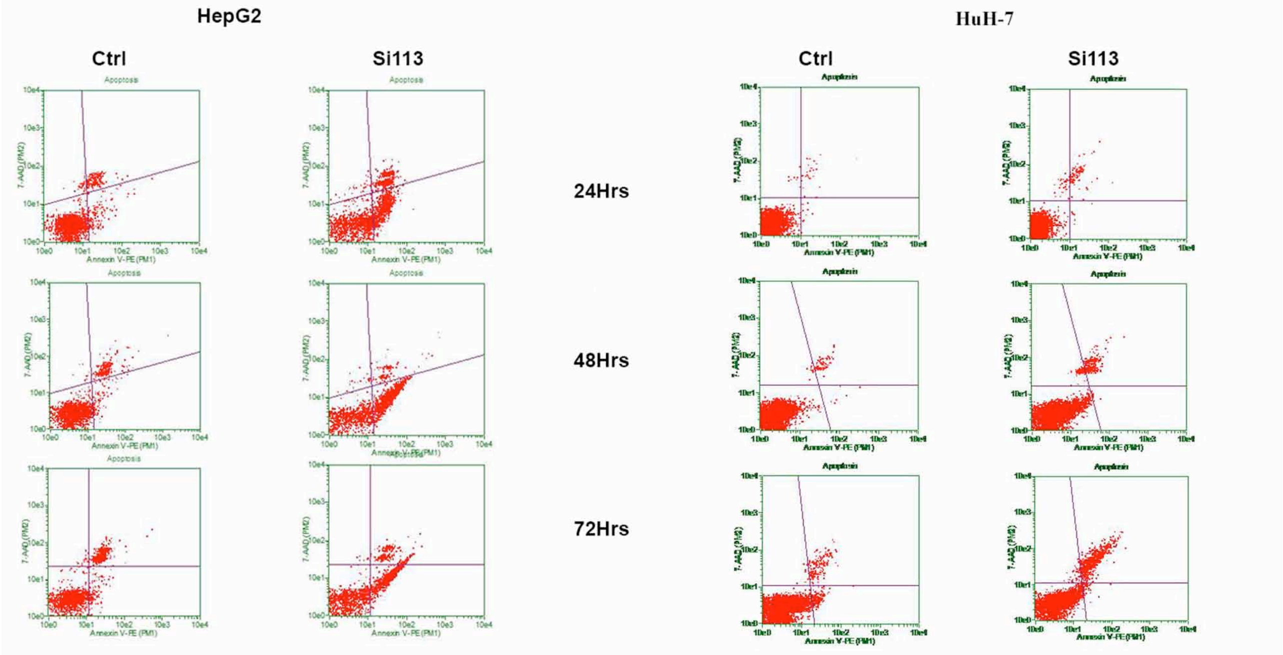

Supplementary Figure S3: Representative histograms of survival analysis with Annexin V/7-AAD of independent triplicate (Figure 2, panel B and D), HepG2 cells (Left) and forHuH-7 cells (right).

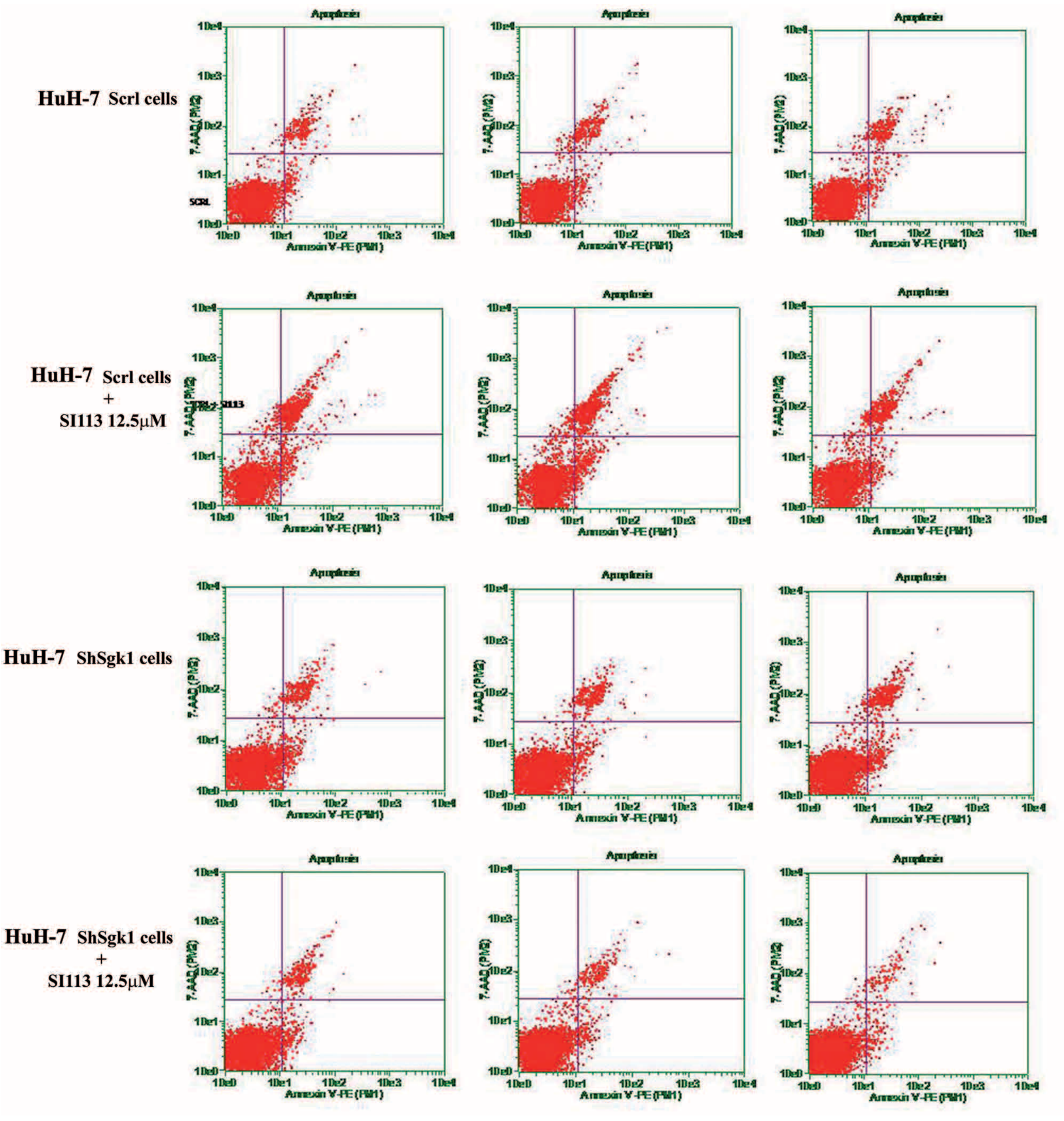

Supplementary Figure S4: Representative histograms of survival analysis with Annexin V/7-AAD of independent triplicates (Figure 3 panel C), for HuH-7 cells (right).

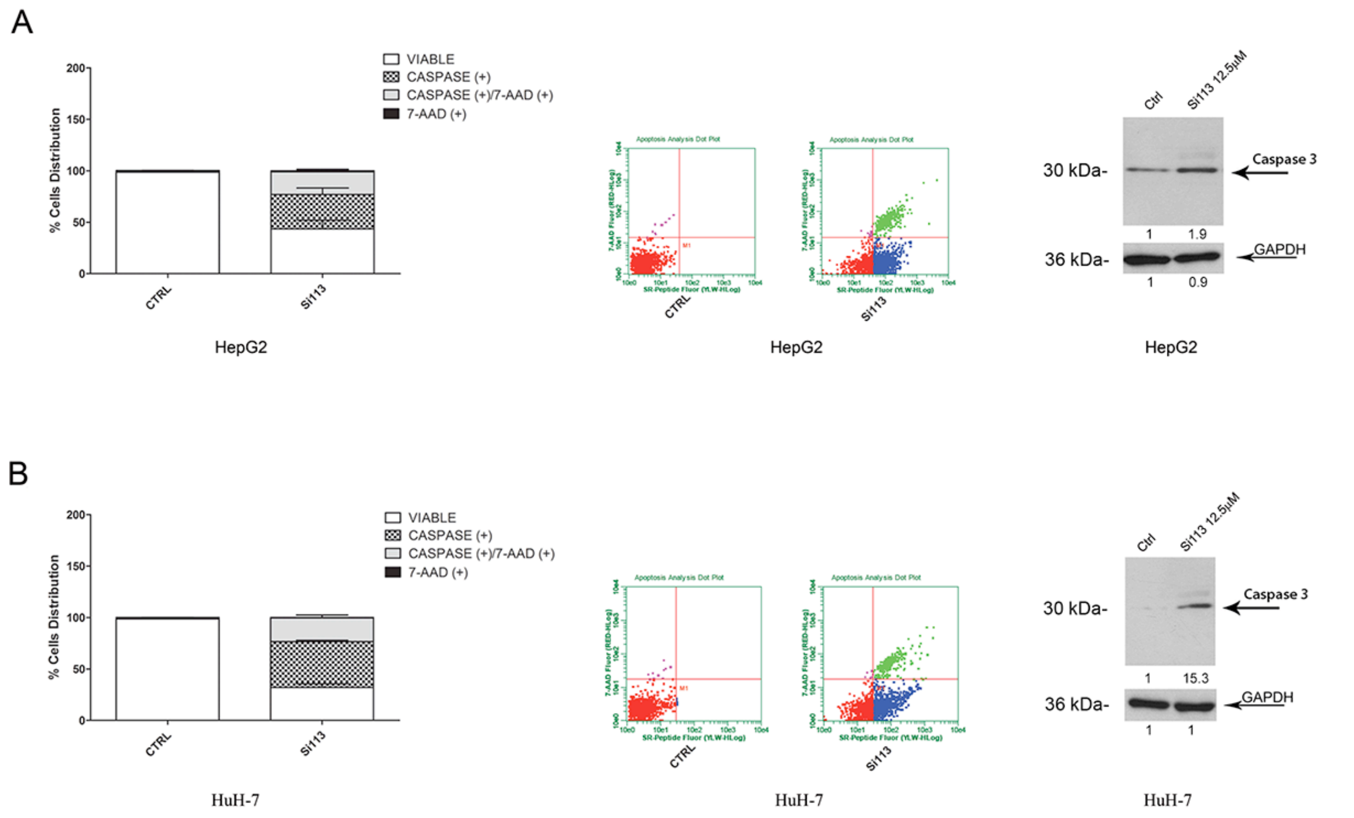

Supplementary Figure S5: HepG2 cell line panel A. and HuH-7cell line panel B. The Histograms represent the percentage of cell distribution treated with SI113 for 72 h analyzed by cytofluorimetry after staining with Guava caspase reagent. A significant increase in apoptotic cells was demonstrated in SI113-treated cells in both HepG2 and HuH-7cultures (Supplementary Figure S5). In particular, in HepG2 cells (panel A), treatment with SI113 for 72 h yielded a significant increase in cells in the middle stages of apoptosis [Caspase Reagent(+) and 7-AAD(−)] (33.3 ± 6.35% after treatment and 0.1 ± 0.05% in control cells) and an increase of cells in the late stages of apoptotic or dead [Caspase Reagent(+) and 7-AAD(+)](22.1 ± 2.28% after treatment and 0.16 ± 0.08% in control cells). In HuH-7cells (panel B) the same treatment yielded increase cells in both stages of middle and late apoptosis (44.6 ± 1.17% and 23.1 ± 2.69% respectively in the treated population vs. 0.26 ± 0.08% and 0.1 ± 0.05% respectively in control samples). The data indicate therefore that SI113 activated the apoptotic response pathways in a highly significant manner. HepG2 cells SI113 12.5 μM caspase(+)/7-AAD(−) $P = 0.006$; caspase(+)/7-AAD(+) $P = 0.0006$. HuH-7cells SI113 12.5 μM caspase(+)/7-AAD(-) $P = 2,99\text{E-}06$; caspase(+)/7-AAD(+) $P = 0,001$.

Label	Accession	Description	Score	Coverage	# Unique Peptides	MW [kDa]	calc. pI	fold change small molecul vs control	±SEM	p value
1	P09651-3	Isoform 2 of Heterogeneous nuclear ribonucleoprotein A1 OS=Homo sapiens GN=HNRNPA1 - [ROA1_HUMAN]	76,57	43,07	9	29,37	9,14	5,82	0,12	0,004263678
2	Q13151	Heterogeneous nuclear ribonucleoprotein A0 OS=Homo sapiens GN=HNRNPA0 PE=1 SV=1 - [ROA0_HUMAN]	9,29	18,36	3	30,82	9,29	5,82	0,09	1,30441E-07
7	Q15102	Platelet-activating factor acetylhydrolase IB subunit gamma OS=Homo sapiens GN=PAFAH1B3 PE=1 SV=1 - [PA1B3_HUMAN]	5,59	11,26	3	25,72	6,84	-50,00	0,05	4,44598E-09
8	P09417	Dihydropteridine reductase OS=Homo sapiens GN=QDPR PE=1 SV=2 - [DHPR_HUMAN]	15,44	30,74	6	25,77	7,37	-50,00	0,10	1,46842E-05
9	P00492	Hypoxanthine-guanine phosphoribosyltransferase OS=Homo sapiens GN=HPRT1 PE=1 SV=2 - [HPRT_HUMAN]	12,61	25,23	5	24,56	6,68	-50,00	0,09	1,30441E-07
10	Q8TAA5	GrpE protein homolog 2, mitochondrial OS=Homo sapiens GN=GRPEL2 PE=1 SV=1 - [GRPE2_HUMAN]	3,80	8,00	2	25,42	7,72	-50,00	0,12	2,78851E-05
11	P25788-2	Isoform 2 of Proteasome subunit alpha type-3 OS=Homo sapiens GN=PSMA3 - [PSA3_HUMAN]	18,38	23,79	7	27,63	5,33	-50,00	0,05	2,73541E-06
12	P78417	Glutathione S-transferase omega-1 OS=Homo sapiens GN=GSTO1 PE=1 SV=2 - [GSTO1_HUMAN]	14,83	21,99	6	27,55	6,60	-50,00	0,07	5,6836E-07
13	P43487	Ran-specific GTPase-activating protein OS=Homo sapiens GN=RANBP1 PE=1 SV=1 - [RANG_HUMAN]	0,00	8,96	2	23,30	5,29	-3,84	0,49	5,45619E-06
15	P61586	Transforming protein RhoA OS=Homo sapiens GN=RHOA PE=1 SV=1 - [RHOA_HUMAN]	13,39	25,91	5	21,75	6,10	-2,04	0,12	3,89707E-06
13	P61106	[illegible] [RAB14_HUMAN]	12,71	35,35	7	23,88	6,21	-2,04	0,09	6,52573E-05
17	O95816	BAG family molecular chaperone regulator 2 OS=Homo sapiens GN=BAG2 PE=1 SV=1 - [BAG2_HUMAN]	12,95	27,49	5	23,76	6,70	1,96	0,03	1,98073E-07
18	P14649	Myosin light chain 6B OS=Homo sapiens GN=MYL6B PE=1 SV=1 - [MYL6B_HUMAN]	8,88	21,15	4	22,75	5,73	1,96	0,06	6,57515E-06
20	P35232	Prohibitin OS=Homo sapiens GN=PHB PE=1 SV=1 - [PHB_HUMAN]	46,62	45,22	11	29,79	5,76	-50,00	0,08	2,89194E-06
21	H0YNE3	Proteasome activator complex subunit 1 OS=Homo sapiens GN=PSME1 PE=4 SV=1 - [H0YNE3_HUMAN]	17,83	29,18	7	26,85	5,78	-50,00	0,10	0,000199821
22	P43487	Ran-specific GTPase-activating protein OS=Homo sapiens GN=RANBP1 PE=1 SV=1 - [RANG_HUMAN]	4,76	8,96	2	23,30	5,29	-50,00	0,13	2,08764E-07
23	O00299	Chloride intracellular channel protein 1 OS=Homo sapiens GN=CLIC1 PE=1 SV=4 - [CLIC1_HUMAN]	55,50	57,68	9	26,91	5,17	50,00	0,01	3,96481E-09
24	P00491	Purine nucleoside phosphorylase OS=Homo sapiens GN=PNP PE=1 SV=2 - [PNPH_HUMAN]	10,39	16,96	4	32,10	6,95	-2,77	0,21	3,30646E-06
25	C9JIM0	MAWD binding protein, isoform CRA_a OS=Homo sapiens GN=PBLD PE=4 SV=1 - [C9JIM0_HUMAN]	4,26	10,36	2	31,39	6,99	-2,77	0,02	1,22249E-07
27	Q14847	LIM and SH3 domain protein 1 OS=Homo sapiens GN=LASP1 PE=1 SV=2 - [LASP1_HUMAN]	1,97	16,86	4	29,70	7,05	2,12	0,11	2,6538E-05
37	Q92905	COP9 signalosome complex subunit 5 OS=Homo sapiens GN=COPS5 PE=1 SV=4 - [CSN5_HUMAN]	4,28	7,19	2	37,55	6,54	-1,88	0,03	0,000557456
38	B4DU58	Macrophage-capping protein OS=Homo sapiens GN=CAPG PE=2 SV=1 - [B4DU58_HUMAN]	3,90	14,37	4	36,23	6,25	-1,88	0,05	1,26902E-06
39	Q9NP79	Vacuolar protein sorting-associated protein VTA1 homolog OS=Homo sapiens	4,65	9,45	2	33,86	6,29	-6,43	0,10	0,057015507
45	P50502	Hsc70-interacting protein OS=Homo sapiens GN=ST13 PE=1 SV=2 - [F10A1_HUMAN]	28,16	26,83	9	41,31	5,27	2,50	0,07	6,05529E-06
46	Q9BYG5	Partitioning defective 6 homolog beta OS=Homo sapiens GN=PARD6B PE=1 SV=1 - [PAR6B_HUMAN]	14,48	22,85	8	41,16	5,58	2,50	0,07	6,80438E-06
47	Q6NXE6-2	Isoform 2 of Armadillo repeat-containing protein 6 OS=Homo sapiens GN=ARMC6 - [ARMC6_HUMAN]	4,67	6,51	2	51,52	6,05	3,00	0,05	2,57318E-07
48	Q8IYS1	Peptidase M20 domain-containing protein 2 OS=Homo sapiens GN=PM20D2 PE=1 SV=2 - [P20D2_HUMAN]	7,74	8,94	3	47,75	5,85	3,00	0,10	8,23499E-06
49	B3KQ59	RuvB-like 2 OS=Homo sapiens GN=RUVBL2 PE=2 SV=1 - [B3KQ59_HUMAN]	4,01	8,85	3	46,28	5,44	3,00	0,08	0,215469075
50	F8VPV9	ATP synthase subunit beta OS=Homo sapiens GN=ATP5B PE=2 SV=1 - [F8VPV9_HUMAN]	20,60	22,39	8	55,27	5,40	2,43	0,07	4,81503E-06
51	P09622	Dihydrolipoyl dehydrogenase, mitochondrial OS=Homo sapiens GN=DLD PE=1 SV=2 - [DLDH_HUMAN]	60,82	32,61	14	54,14	7,85	-50,00	0,09	4,30675E-05
52	P14618	Pyruvate kinase isozymes M1/M2 OS=Homo sapiens GN=PKM PE=1 SV=4 - [KPYM_HUMAN]	29,08	32,02	13	57,90	7,84	-50,00	0,12	1,42702E-07
53	Q9Y3I0	tRNA-splicing ligase RtcB homolog OS=Homo sapiens GN=C22orf28 PE=1 SV=1 - [RTCB_HUMAN]	28,40	25,94	12	55,17	7,23	-50,00	0,12	0,000418818
54	P00367	Glutamate dehydrogenase 1, mitochondrial OS=Homo sapiens GN=GLUD1 PE=1 SV=2 - [DHE3_HUMAN]	24,32	24,55	11	61,36	7,80	-50,00	0,05	5,39616E-09
55	P00352	Retinal dehydrogenase 1 OS=Homo sapiens GN=ALDH1A1 PE=1 SV=2 - [AL1A1_HUMAN]	21,35	24,35	10	54,83	6,73	-50,00	0,12	1,42702E-07
56	P28838-2	Isoform 2 of Cytosol aminopeptidase OS=Homo sapiens GN=LAP3 - [AMPL_HUMAN]	9,07	12,50	5	52,74	6,74	-50,00	0,09	0,04778796
57	P14868	Aspartate--tRNA ligase, cytoplasmic OS=Homo sapiens GN=DARS PE=1 SV=2 - [SYDC_HUMAN]	7,94	8,18	4	57,10	6,55	-50,00	0,03	2,54327E-07
58	Q9UMS4	Pre-mRNA-processing factor 19 OS=Homo sapiens GN=PRPF19 PE=1 SV=1 - [PRP19_HUMAN]	7,76	8,93	4	55,15	6,61	-50,00	0,05	0,000105914
59	P78371	T-complex protein 1 subunit beta OS=Homo sapiens GN=CCT2 PE=1 SV=4 - [TCPB_HUMAN]	175,07	76,45	35	57,45	6,46	-50,00	0,02	3,12512E-08
60	P28838-2	Isoform 2 of Cytosol aminopeptidase OS=Homo sapiens GN=LAP3 - [AMPL_HUMAN]	29,86	30,12	12	52,74	6,74	-50,00	0,06	2,82944E-06
61	O43175	D-3-phosphoglycerate dehydrogenase OS=Homo sapiens GN=PHGDH PE=1 SV=4 - [SERA_HUMAN]	40,04	29,08	12	56,61	6,71	-50,00	0,07	8,79575E-06
62	G5EA52	Protein disulfide isomerase family A, member 3, isoform CRA_b OS=Homo sapiens GN=PDIA3 PE=2 SV=1 - [G5EA52_HUMAN]	24,52	24,54	11	54,93	6,86	-50,00	0,01	1,61898E-08
63	P13674	Prolyl 4-hydroxylase subunit alpha-1 OS=Homo sapiens GN=P4HA1 PE=1 SV=2 - [P4HA1_HUMAN]	87,91	50,75	1	61,01	6,01	-50,00	0,07	3,75129E-06
64	Q13177	Serine/threonine-protein kinase PAK 2 OS=Homo sapiens GN=PAK2 PE=1 SV=3 - [PAK2_HUMAN]	32,50	29,01	15	58,01	5,96	-50,00	0,21	1,30385E-06
66	P02787	Serotransferrin OS=Homo sapiens GN=TF PE=1 SV=3 - [TRFE_HUMAN]	202,16	63,47	41	77,01	7,12	-50,00	0,11	4,47592E-05
67	Q96RQ3	Methylcrotonoyl-CoA carboxylase subunit alpha, mitochondrial OS=Homo sapiens GN=MCCC1 PE=1 SV=3 - [MCCA_HUMAN]	36,71	24,69	13	80,42	7,78	-50,00	0,05	2,52066E-06
68	O00429-2	Isoform 4 of Dynamin-1-like protein OS=Homo sapiens GN=DNM1L - [DNM1L_HUMAN]	10,95	11,59	6	80,49	6,71	-50,00	0,07	0,03789384
69	Q5JQ13	Vinculin (Fragment) OS=Homo sapiens GN=VCL PE=4 SV=1 - [Q5JQ13_HUMAN]	5,81	7,57	5	87,39	5,82	-50,00	0,04	2,51939E-05
70	Q8WUM4	Programmed cell death 6-interacting protein OS=Homo sapiens GN=PDCD6IP PE=1 SV=1 - [PDC6I_HUMAN]	12,86	6,22	5	95,96	6,52	-50,00	0,02	2,19268E-05
71	B9A067	Mitochondrial inner membrane protein OS=Homo sapiens GN=IMMT PE=2 SV=2 - [B9A067_HUMAN]	23,24	14,63	8	78,92	7,06	-50,00	0,07	3,40219E-06
72	Q96RP9	Elongation factor G, mitochondrial OS=Homo sapiens GN=GFM1 PE=1 SV=2 - [EFGM_HUMAN]	11,12	11,58	8	83,42	7,01	-50,00	0,06	1,28659E-07
73	Q99714-2	Isoform 2 of 3-hydroxyacyl-CoA dehydrogenase type-2 OS=Homo sapiens GN=HSD17B10 - [HCD2_HUMAN]	22,14	48,81	7	25,97	7,21	-50,00	0,09	4,11749E-06
74	P22061	Protein-L-isoaspartate(D-aspartate) O-methyltransferase OS=Homo sapiens GN=PCMT1 PE=1 SV=4 - [PIMT_HUMAN]	14,44	23,35	5	24,62	7,21	-50,00	0,09	0,107734538
75	P62826	GTP-binding nuclear protein Ran OS=Homo sapiens GN=RAN PE=1 SV=3 - [RAN_HUMAN]	6,79	19,44	4	24,41	7,49	-50,00	0,08	2,40752E-06
76	P62826	GTP-binding nuclear protein Ran OS=Homo sapiens GN=RAN PE=1 SV=3 - [RAN_HUMAN]	50,97	48,61	9	24,41	7,49	-50,00	0,10	2,15337E-05
77	Q6P587	Acylpyruvase FAHD1, mitochondrial OS=Homo sapiens GN=FAHD1 PE=1 SV=2 - [FAHD1_HUMAN]	7,89	16,52	3	24,83	7,39	-50,00	0,13	7135104,709
78	Q9UL25	Ras-related protein Rab-21 OS=Homo sapiens GN=RAB21 PE=1 SV=3 - [RAB21_HUMAN]	4,49	9,33	2	24,33	7,94	-50,00	0,12	0,000209409
79	O95372	Acyl-protein thioesterase 2 OS=Homo sapiens GN=LYPLA2 PE=1 SV=1 - [LYPA2_HUMAN]	8,75	29,00	5	24,72	7,23	8,95	0,06	2,69808E-09
80	P30042-2	Isoform Short of ES1 protein homolog, mitochondrial OS=Homo sapiens GN=C21orf33 - [ES1_HUMAN]	6,83	16,46	3	24,74	8,13	8,95	0,12	7,1351E-08
81	P30043	Flavin reductase (NADPH) OS=Homo sapiens GN=BLVRB PE=1 SV=3 - [BLVRB_HUMAN]	2,17	11,65	2	22,11	7,65	8,95	0,21	3,40219E-06
82	P30085	UMP-CMP kinase OS=Homo sapiens GN=CMPK1 PE=1 SV=3 - [KCY_HUMAN]	91,11	69,39	17	22,21	5,57	-50,00	0,05	7,1351E-08
83	P09211	Glutathione S-transferase P OS=Homo sapiens GN=GSTP1 PE=1 SV=2 - [GSTP1_HUMAN]	170,49	62,86	14	23,34	5,64	-50,00	0,08	5,29569E-05
84	Q9NRV9	Heme-binding protein 1 OS=Homo sapiens GN=HEBP1 PE=1 SV=1 - [HEBP1_HUMAN]	26,63	50,26	7	21,08	5,80	-50,00	0,04	1,87565E-06
85	Q9NXU5	ADP-ribosylation factor-like protein 15 OS=Homo sapiens GN=ARL15 PE=1 SV=1 - [ARL15_HUMAN]	6,23	14,71	3	22,86	5,63	-50,00	0,08	1,26033E-06
86	P49721	Proteasome subunit beta type-2 OS=Homo sapiens GN=PSMB2 PE=1 SV=1 - [PSB2_HUMAN]	14,09	27,86	5	22,82	7,02	-50,00	0,08	1,65323E-06
87	P37802	Transgelin-2 OS=Homo sapiens GN=TAGLN2 PE=1 SV=3 - [TAGL2_HUMAN]	12,17	34,17	6	22,38	8,25	-50,00	0,11	6,3451E-07

Supplementary Figure S6: List of the proteins consistently up- or down-regulated in HuH-7 cell line treated with 12.5 μM SI113 compared with control counterpart.

ID	Molecules in Network	Score	Focus Molecules	Top Diseases and Functions
1	20s proteasome,Actin,ALDOA,Alpha tubulin,ATP5B,BAG2,CCDC50,CCT2,CCT3,COPS5,DARS,DNM1L,ERK1/2,F Actin,HNRNPA0,Hsp90,HSPB1,Immunoproteasome Pa28/20s,LAP3,LASP1,PCMT1,PDCD6IP,PGAM1,PKM,PRPF19,PSMA3,PSMB2,PSMC2,PSME1,QDPR,RTCB,TF,TPI1,Ubiquitin,VCL	63	27	Carbohydrate Metabolism, Energy Production, Nucleic Acid Metabolism
2	AKR7A2,Akt,Ap1,caspase,CD3,Creb,DNAJB11,FSH,GLUD1,GOT1,HBB,HNRNPA1,HPRT1,Hsp70,LDL,Lh,Mlc,MYL6B,p85 (pik3r),PAFAH1B3,PAK2,PCBP1,PDGF BB,PDLIM1,PHB,PHGDH,PNP,RAB14,RAN,RANBP1,RHOA,RNA polymerase II,ST13,trypsin,Vegf	39	20	Amino Acid Metabolism, Small Molecule Biochemistry, Cellular Growth and Proliferation
3	AK1,ANKRD27,ARL15,ARMC6,C21orf33/LOC102724023,CLN3,CMPK1,CRMP1,EIF2AK4,EMC2,EXOSC4,FAHD1,FUNDC2,GFM1,GRIPAP1,GRPEL2,HEBP1,MCCC1,NCCRP1,PBLD,PDE6D,PM20D2,PPAP2A,PSPH,RAB21,RABGEF1,RPIA,RPL8,RPL9,RSL1D1,TFG,TUBB2A,UBC,VPS4B,VTA1	32	16	Cellular Assembly and Organization, Developmental Disorder, Hereditary Disorder
4	ACADM,ALDH1A1,B3GNT5,BLVRB,BRDT,CAPG,CD55,CLIC1,ERK,ETFB,GSTA2,GSTM2,GSTP1,GYPC,Histone h3,Histone h4,Immunoglobulin,Insulin,Jnk,mannose,MTORC1,NFkB (complex),P38 MAPK,P4HA1,PDE1C,PDIA3,PI3K (complex),Pka,Pkc(s),RUVBL2,SIRT4,sphingomyelinase,TAOK2,TMOD2,ZNF302	18	10	Drug Metabolism, Glutathione Depletion In Liver, Auditory Disease
5	ARMCX3,C10orf88,CHCHD6,DLD,DNAJC11,EHD2,GLDC,GSTM2,Gstm3,GSTO1,HSD17B10,IMMT,IPMK,KIF22,LYPLA2,MRPL44,MTX1,MTX2,MYO19,NDUFB9,NIPSNAP3A,OGDH,PARD6B,PDHX,PLA2G15,PPOX,RER1,SAMM50,SDHAF2,SERPINB1,STK17A,TAGLN2,TRMT10C,TXNDC9,UBC	13	8	Developmental Disorder, Hereditary Disorder, Metabolic Disease

Supplementary Figure S7: IPA analysis prediction for 5 networks of interacting protein clusters according to the identifiers' HomoloGene to the ortholog information in the Ingenuity Knowledge Base (IKB).

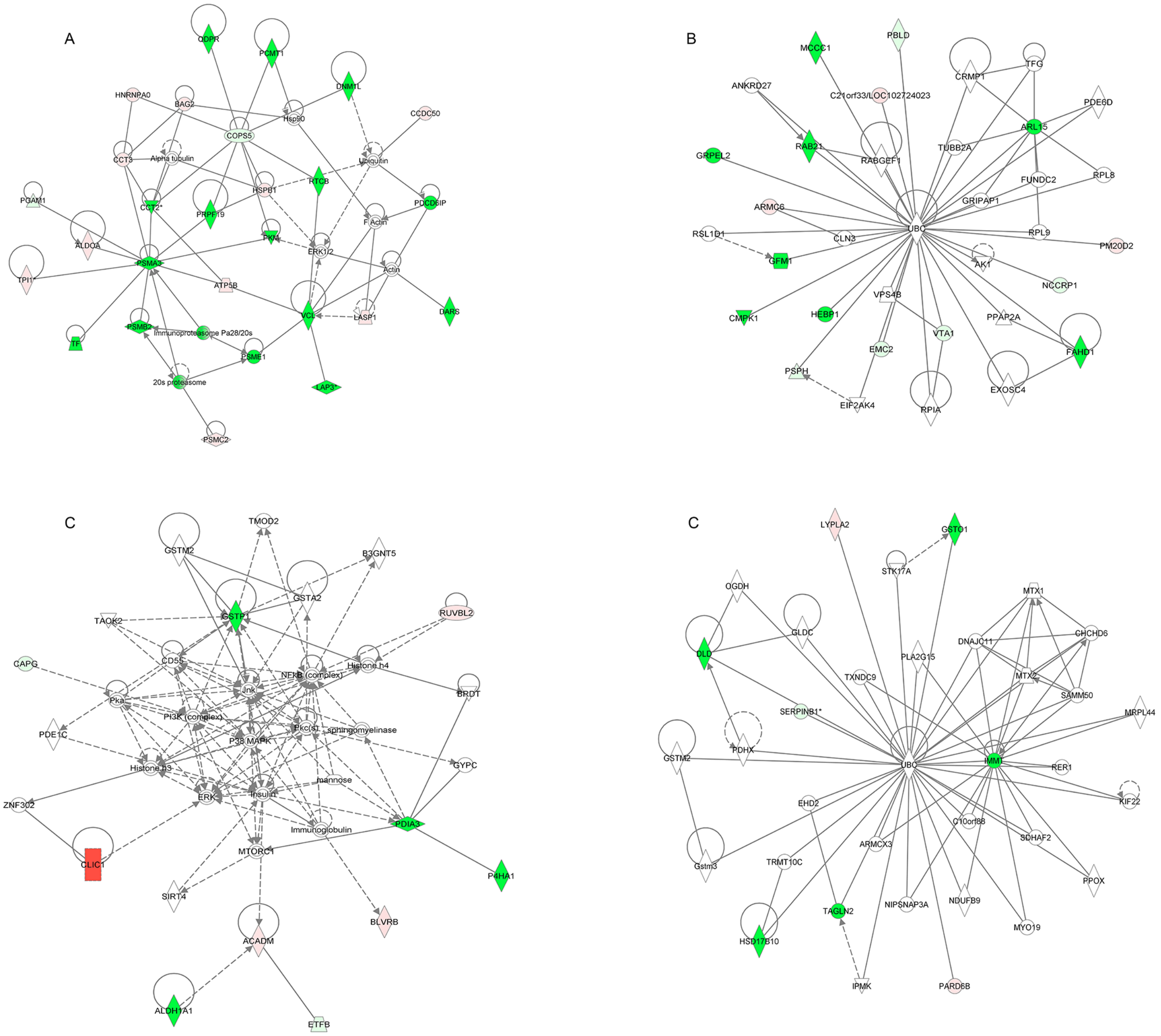

Supplementary Figure S8: Graphical representation of pathways clustered for functional elements as identified in proteomic analysis and analyzed in the Ingenuity Knowledge Base (IKB).

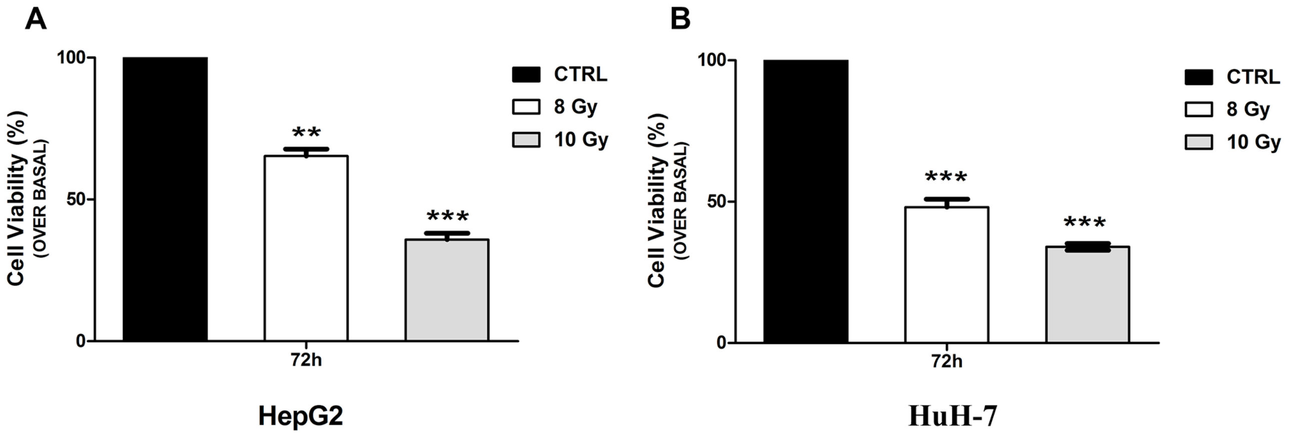

Supplementary Figure S9: HepG2 cell line panel A. and HuH-7cell line panel B. The histograms represent the number of cells treated for 72 h with respectively 8 and 10 Gy of radiation. Results are expressed as percentage of the number of control cells (HepG2 Ctrl 89333 ± 667; HuH-7 Ctrl 156693 ± 3266). Values for treated and untreated cells in the different conditions were compared by unpaired two-tailed Student's *t* test. Results represent the mean ± S.D. of three independent experiments for each cell line. Statistical significance: HepG2 cells. 8 Gy vs. ctrl cells $P = 0.004$, 10 Gy vs. ctrl cells $P = 0.001$, 10 Gy vs. 8 Gy $P = 0.004$. HuH-7 cells. 8 Gy vs. ctrl cells $P = 1.8^{-5}$, 10 Gy vs. ctrl cells $P = 3.53^{-5}$, 10 Gy vs. 8 Gy $P = 0.0006$.

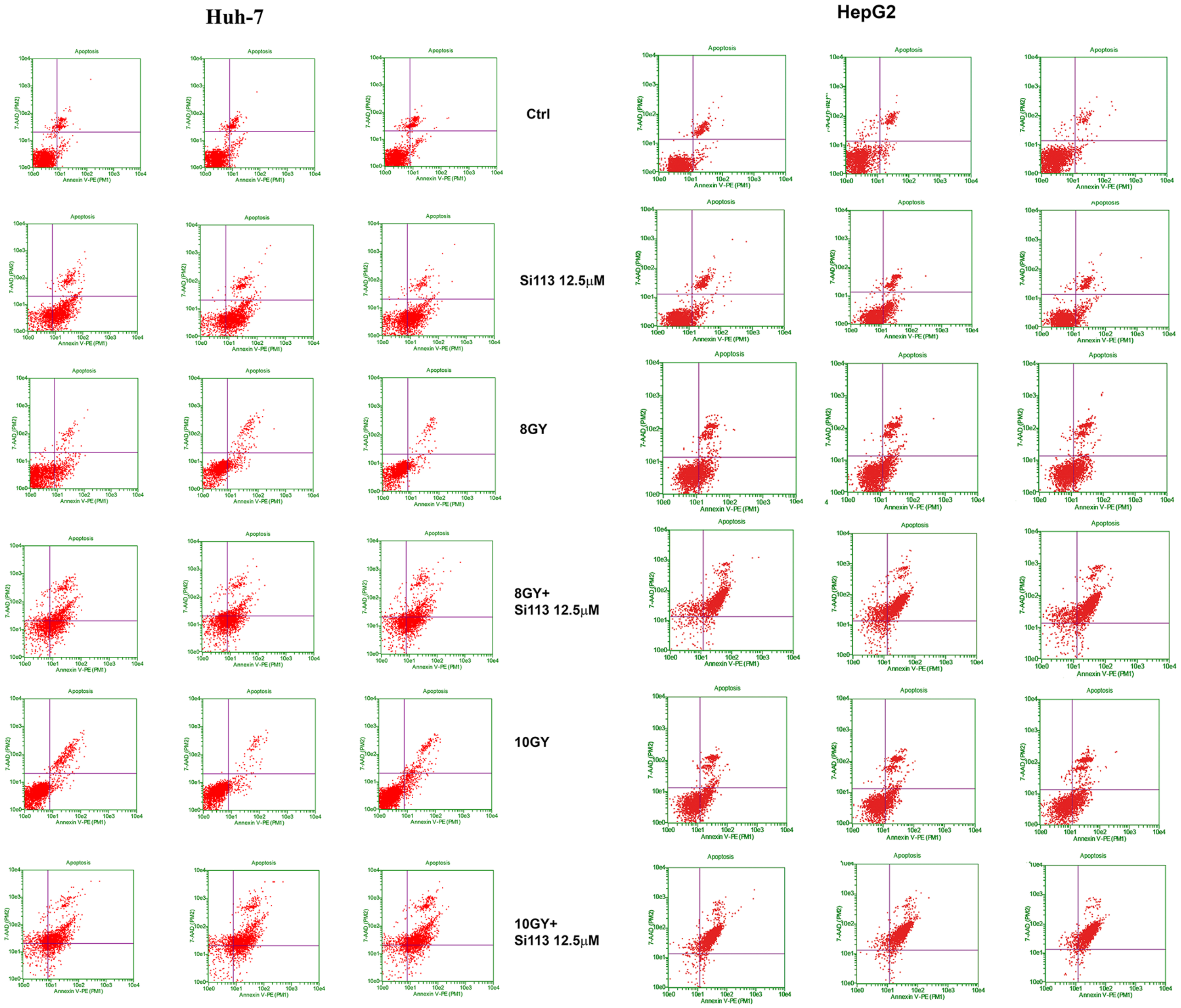

Supplementary Figure S10: All histograms related to the apoptosis/necrosis analysis for HepG2 cell line (right panel) and HuH-7 cell line (left panel) in Figure 6 panel C.
